# Yorkshire Lung Screening Trial (YLST) pathway navigation study: a protocol for a nested randomised controlled trial to evaluate the effect of a pathway navigation intervention on lung cancer screening uptake

**DOI:** 10.1136/bmjopen-2024-084577

**Published:** 2024-07-09

**Authors:** Daisy McInnerney, Irene Simmonds, Neil Hancock, Suzanne Rogerson, Jason Lindop, Rhian Gabe, Daniel Vulkan, Catriona Marshall, Philip A J Crosbie, Matthew E J Callister, Samantha L Quaife

**Affiliations:** 1 Wolfson Institute of Population Health, Queen Mary University, London, UK; 2 Leeds Institute of Health Sciences, University of Leeds, Leeds, UK; 3 Department of Research and Innovation, Leeds Teaching Hospitals NHS Trust, Leeds, UK; 4 Division of Infection, Immunity and Respiratory Medicine, The University of Manchester, Wythenshawe, UK; 5 Department of Respiratory Medicine, Leeds Teaching Hospitals NHS Trust, Leeds, UK

**Keywords:** Patient Navigation, Respiratory tract tumours, Mass Screening, Health Equity, Lung Diseases

## Abstract

**Introduction:**

Lung cancer is the most common cause of cancer death globally. In 2022 the UK National Screening Committee recommended the implementation of a national targeted lung cancer screening programme, aiming to improve early diagnosis and survival rates. Research studies and services internationally consistently observe socioeconomic and smoking-related inequalities in screening uptake. Pathway navigation (PN) is a process through which a trained pathway navigator guides people to overcome barriers to accessing healthcare services, including screening. This nested randomised controlled trial aims to determine whether a PN intervention results in more individuals participating in lung cancer screening compared with the usual written invitation within a previous non-responder population as part of the Yorkshire Lung Screening Trial (YLST).

**Methods and analysis:**

A two-arm randomised controlled trial and process evaluation nested within the YLST. Participants aged 55–80 (inclusive) who have not responded to previous postal invitations to screening will be randomised by household to receive PN or usual care (a further postal invitation to contact the screening service for a lung health check) between March 2023 and October 2024. The PN intervention includes a postal appointment notification and prearranged telephone appointment, during which a pathway navigator telephones the participant, following a four-step protocol to introduce the offer and conduct an initial risk assessment. If eligible, participants are invited to book a low-dose CT (LDCT) lung cancer screening scan. All pathway navigators receive training from behavioural psychologists on motivational interviewing and communication techniques to elicit barriers to screening attendance and offer solutions.

**Coprimary outcomes:**

The number undergoing initial telephone assessment of lung cancer risk. The number undergoing an LDCT screening scan.

Secondary outcomes include demographic, clinical and risk parameters of people undergoing telephone risk assessment; the number of people eligible for screening following telephone risk assessment; the number of screen-detected cancers diagnosed; costs and a mixed-methods process evaluation.

Descriptive analyses will be used to present numbers, proportions and quantitative components of the process evaluation. Primary comparisons of differences between groups will be made using logistic regression. Applied thematic analysis will be used to interpret qualitative data within a conceptual framework based on the COM-B framework. A health economic analysis of the PN intervention will also be conducted.

**Ethics and dissemination:**

The study is approved by the Greater Manchester West Research Ethics Committee (18-NW-0012) and the Health Research Authority following the Confidentiality Advisory Group review. Results will be shared through peer-reviewed scientific journals, conference presentations and on the YLST website.

**Trial registration numbers:**

ISRCTN42704678NCT03750110ISRCTN42704678 and NCT03750110.

**STRENGTHS AND LIMITATIONS OF THIS STUDY**

- Robust theory and evidence-based intervention development methods are used to inform the novel, behavioural-science-informed pathway navigation intervention tailored to the UK lung cancer screening context.
- A prospective randomised evaluation of the effects of pathway navigation on participation rates in lung cancer risk assessment and community-based LDCT lung cancer screening scans among previous non-responders to provide gold-standard confirmatory evidence and cost-effectiveness estimation, which will inform the optimal approach for screening implementation.
- The service demonstration study design, nested within the Yorkshire Lung Screening Trial (YLST), enhances ecological validity, providing a realistic indication of uptake following pathway navigation in a real-world clinical context.
- The pathway navigation intervention was introduced late in the YLST participant screening journey (after two rounds of non-response to invitations across 4 years); if implemented in practice it is more likely this would be deployed following non-response to one round of screening invitations.

## Introduction

 Lung cancer is the most common cause of cancer death globally, causing approximately 35 000 deaths per year in the UK.^1^ The low survival rate (around 85% of people die within 5 years of a diagnosis^1^) is largely attributed to the majority of people being diagnosed at a late stage when treatment is less likely to be curative.^2^ Survival rates are lower still for people experiencing socioeconomic deprivation^3^ yet their incidence rates are highest.^4^ Globally, trials of lung cancer screening using low-dose CT scanning (LDCT) to detect asymptomatic, early-stage disease, have demonstrated a reduction in lung cancer mortality compared with controls in high-risk populations (adults over 55 with a smoking history).^5^,^11^ A meta-analysis of nine trials found LDCT screening was associated with a 16% relative reduction in lung cancer mortality compared with non-screened controls.^11^ Based on this combined evidence, the UK National Screening Committee has recommended implementation of a nationwide targeted lung cancer screening programme.^2^ This programme is being rolled out across England via the expansion of the Targeted Lung Health Check programme, which currently offers LDCT lung cancer screening to people at high risk of lung cancer.

While lung cancer screening offers great promise for reducing lung cancer deaths, care must be taken not to widen the existing lung cancer mortality deprivation gap.^3^ Socioeconomic and smoking-related inequalities in uptake have consistently been observed across trials and services internationally, with uptake significantly lowest among people living in the most deprived areas, and those who currently smoke.^12^,^16^ There is also emerging evidence of lower participation by ethnic minority groups, such as those of white ethnicity other than British.^16^ This means those communities with the highest lung cancer incidence are least likely to be screened, reducing both the equity and effectiveness of screening. The ongoing Yorkshire Lung Screening Trial (YLST), which invites adults with a smoking history aged 55–80 to a telephone-based risk assessment to determine eligibility for a Lung Health Check screening appointment (subsequently offered at a mobile unit), found just 50.8% of those invited took up the offer of the telephone assessment in the first round of screening.^17^ Crucially, uptake was skewed towards those from more affluent areas, and those people who had quit smoking (compared with those who continue to smoke). This was despite the use of evidence-based interventions from other screening programmes, including general practitioner (GP) endorsement, a low-burden leaflet and reminder invitations.^18^,^23^ These figures compare favourably to other UK trials and pilots, which have a 20.4%–52.6% range in response.^23^,^25^

Pathway navigation (PN), sometimes referred to as patient navigation, aims to help individuals overcome barriers to early and effective diagnosis and treatment, particularly among populations who experience inequalities in cancer outcomes. It can be split into three phases: (1) navigation to screening, (2) diagnostic evaluation and (3) treatment.^26^ Research in the USA suggests navigation programmes are effective at improving participation in different types of cancer screening^27^ and an American Thoracic Society statement^28^ concluded navigation should be integrated within lung cancer screening programmes as a strategy to reduce disparities in uptake. In a randomised trial (n=1200) comparing navigators with usual care among a lower socioeconomic population, uptake was significantly improved in the navigator arm (23.5%) relative to usual care (8.6%).^26^

To date, there have been no published studies specifically testing PN for individuals invited to lung cancer screening in the UK. One small feasibility study tested whether navigation by specialist screening practitioners could improve the uptake of bowel scope screening.^29^ However, in that context, it was not feasible to successfully implement the intervention as telephone numbers were not available from health records and participants had to explicitly consent to take part in the study before providing their telephone number to receive navigation. The authors reflected this resulted in a selection bias, whereby only participants engaged in the screening process (and arguably those least likely to require navigation) would consent to take part. However, many cancer alliances in the UK (which do not require consent prior to patient contact) have demonstrated the feasibility of using telephone-based interventions to improve the uptake of cancer screening where numbers are available. For example, a case study from West London RM Partners Cancer Alliance reports successful contact of 13 000 individuals by telephone of which 25% subsequently participated in bowel cancer screening.^30^

While the potential of PN to reach high-risk non-responder populations is promising, adoption of this type of intervention within a national screening programme in the UK would involve considerable resources. It is, therefore, vital that its effect is analysed within the UK health system to demonstrate efficacy and cost-effectiveness. Likewise, it is crucial to understand any barriers to intervention efficacy that may lay in implementation and service design. The YLST recently received funding for a third round of screening to continue the provision of biennial Lung Health Checks. This offers an opportunity to further develop an understanding of barriers to participation and to test if PN can maximise engagement in a lung cancer screening programme in the UK context.

Therefore, the aim of the study is to determine whether PN results in more individuals participating in the telephone risk assessment and, if eligible, the LDCT screening scan as part of their Lung Health Check, compared with usual written invitation within a previous non-responder population. In addition, a mixed-methods process evaluation with people who have repeatedly not responded to invitation will provide an avenue to understand issues preventing individuals from considering or being able to take part in LDCT lung cancer screening, as well as how PN might work, its acceptability and how it could be protocolised at scale, to inform screening invitation and delivery.

## Methods and analysis

### Study design

**Figure 1:**
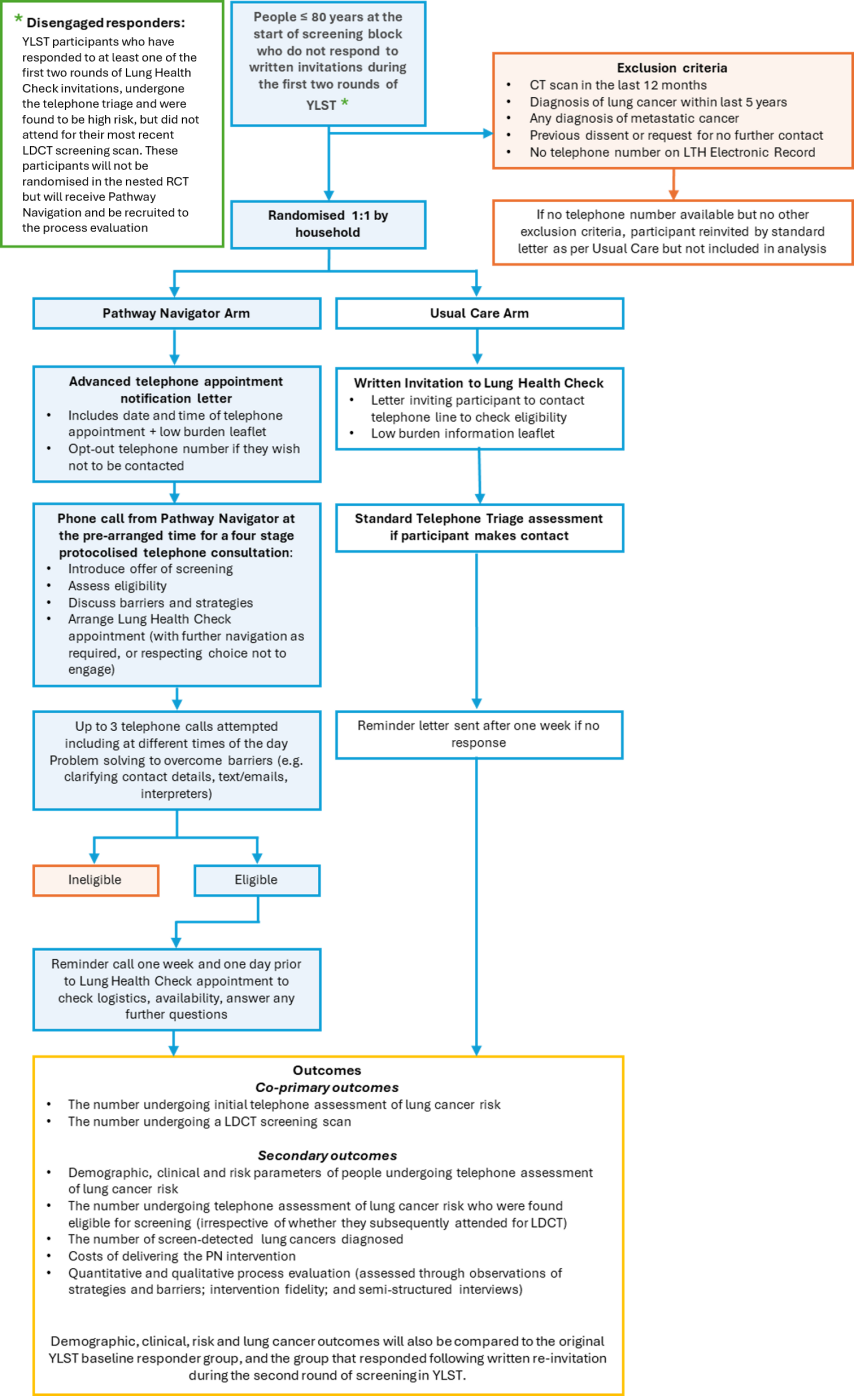
YLST pathway navigation study flow chart. COM-B, capability opportunity motivation-behaviour; GP, general practitioner; LDCT, low-dose CT; LTH, Leeds Teaching Hospitals; YLST, Yorkshire Lung Screening Trial.

A two-arm randomised controlled trial (RCT) with mixed-methods process evaluation nested within the YLST. The methods of the YLST are published elsewhere.^18^ The study flow is described in figure 1. The protocol is reported in line with the Standard Protocol Items: Recommendations for Interventional Trials (SPIRIT) statement (online supplemental file 1).^31^

### Outcomes

For the nested RCT, the outcomes are stated below. These outcomes will also be reported for the process evaluation.

### Coprimary outcomes

1. The number undergoing initial telephone assessment of lung cancer risk.
2. The number undergoing a LDCT screening scan.

### Secondary outcomes

- Demographic, clinical and risk parameters of people undergoing telephone assessment of lung cancer risk.
- The number undergoing telephone assessment of lung cancer risk who were found eligible for screening (irrespective of whether they subsequently attended for LDCT).
- The number of screen-detected lung cancers diagnosed.
- Costs of delivering the PN intervention.
- Quantitative and qualitative process evaluation (assessed through observations of strategies and barriers; acceptability; intervention fidelity; and semistructured interviews).

Demographic, clinical, risk and lung cancer outcomes will also be compared with the original YLST baseline responder group, and the group that responded following written reinvitation during the second round of screening in YLST.

### Participants

**Repeat non-responders (nested RCT and process evaluation participants):** Participants for the RCT will be those who have not responded to a postal invitation to participate in a telephone assessment of lung cancer risk (Lung Health Check eligibility) within the YLST, either as part of the initial invitation round or after being reinvited approximately 2 years later.

**Disengaged responders (process evaluation participants):** Participants in this group will not be randomised within the nested RCT but will all receive PN and be recruited to the process evaluation. This group is made up of YLST participants who have responded to at least one of the first two rounds of Lung Health Check invitations, undergone the telephone triage and were found to be high risk, but did not attend for their most recent LDCT screening scan. This includes people first found to be eligible through telephone assessment of lung cancer risk during:

1. The first screening round, those who attended their first scan, remained eligible at their subsequent eligibility reassessment during the second round but did not attend for their second LDCT screening scan appointment.
2. The second screening round but did not attend their LDCT screening scan appointment.

The group does not include people who were risk assessed during the first screening round, found to be eligible, but did not attend their first scan (since they were already recontacted during the second round).

### Eligibility criteria

For the nested RCT, the full eligibility criteria are stated below. Eligibility criteria for the process evaluation are as described above, including both repeat non-responders and disengaged responders.

#### Inclusion criteria

- Aged 55–80 (inclusive) at the time of the planned screening block (all non-responding participants will be assigned an intended screening block between March 2023 and October 2024; participant age will be calculated on the first day of screening during that block to determine eligibility).
- Assigned to the intervention group (lung cancer screening arm) of YLST.
- No previous YLST telephone triage assessment.

#### Exclusion criteria

- Previous registration of dissent from data usage or further contact from YLST team.
- National data opt-out was recorded prior to the screening round.
- Lung cancer diagnosis within 5 years prior to the screening round.
- Any metastatic cancer diagnosis.
- No telephone number on Leeds Teaching Hospitals Electronic Patient Record.

### Randomisation

Repeat non-responders will be randomised to PN or usual care (control) by household so that cohabiting individuals are assigned to the same arm of the study to avoid contamination. Randomisation will take place after completion of the second round of screening but before reinvitation to the third round of screening. All disengaged responders will receive PN (ie, no randomisation) and will not be randomised as part of the nested RCT, to avoid contaminating the sample (since disengaged responders represent a different subpopulation to repeat non-responders).

### Sample size

#### Nested RCT

The sample size for the RCT is calculated based on the number of people who have not responded to the invitation during the first screening round of YLST. Of the 22 128 people who did not respond during the baseline round, 20 074 people were aged ≤76 years at the time of original GP data extraction (and thus would be aged ≤80 years at the time of proposed PN randomisation) and remained alive at the time of designing the PN study. After accounting for an anticipated 10% response rate to written invitation during the second round, we estimated 18 067 potentially eligible participants for PN randomisation, of which 74.1% had contact numbers recorded, leaving a total eligible population of 13 388.

An 8% response rate to written invitations is anticipated in the control arm, increasing to 12% in the PN arm, based on previous randomised navigator trials in lower socioeconomic populations.^26^ Baseline data from YLST indicate approximately 35% of telephone responders were deemed high risk. Assuming a similar rate in the repeat non-responders in this nested PN substudy and further, considering that only 84% of those assessed as high risk had an LDCT scan (as was observed during the first round of screening in YLST), would infer an increase from 2.4% (standard invitation arm) to 3.5% (PN arm) of those invited responding, being high risk and receiving an LDCT scan. Based on these assumptions, and with an anticipated 13 388 participants, as can be seen from the sample size calculation provided in table 1, there would be ample power (>90%) to detect an increase in both response with telephone assessment and attendance in terms of undergoing an LDCT scan using two-sided tests at the 2.5% significance level to account for coprimary outcomes and using a continuity correction, and adjusting for centre effects by household as appropriate.

**Table 1:** Sample size considerations for coprimary outcomes in the nested RCT

Outcome	Comparisons of expected percentage of those randomised: control versus navigator intervention	90% power, two-sided test at 5% significance level, continuity corrected. Numbers are required.	90% power, two-sided test with alpha of 0.025 to account for coprimary outcomes*, continuity corrected. Numbers are required.
Completed telephone risk assessment	8% vs 12%	2460 (1230 per group)	2888 (1444 per group)
Eligible responders(assumes 35% responders eligible)	2.8% vs 4.2%	7522 (3761 per group)	
Eligible responders having a scan(assumes 84% of eligible have a scan, based on 6650/7958 in YLST first round)	2.4% vs 3.5%	10 302 (5151 per group)	12 104 (6052 per group)

* Co-primary outcomes: (1) The number undergoing initial telephone assessment of lung cancer risk; (2) The number undergoing an LDCT screening scan.

RCTrandomised controlled trialYLSTYorkshire Lung Screening Trial

#### Process evaluation

The sample size for each component of the process evaluation is as follows:

- Quantitative evaluation of barriers and strategies to undergoing a risk assessment and/or attending a screening appointment discussed during PN calls. These will be recorded using a standardised observation proforma (online supplemental file 2) after every PN telephone call (up to five per participant) and after every telephone triage telephone appointment conducted within the control arm.
- Semistructured interviews (n<60): Purposive sampling will be used to recruit a diverse sample of participants who have undergone the PN intervention with respect to demographic characteristics, smoking status and intention to attend a lung screening appointment. Up to 30 participants will be interviewed within each group (ie, non-responders and disengaged), in line with norms for qualitative research, and to ensure adequate representation of those subsequently attending and declining a screening appointment.
- Fidelity observations: A subset of 200 PN calls will be observed. The number has been selected to ensure variability in the calls being observed (with regard to screening day, team member delivering the call, etc) while being possible to conduct within the time frame and in line with other similar studies of intervention fidelity.^32^

### Intervention

#### Development

A theory-and-evidence-based intervention development process was followed.^33^ The navigation-to-screening manual was informed by a bowel screening navigation intervention.^34^ Strategies and barriers were based on existing research investigating factors influencing lung cancer screening uptake^1535^,^38^ and through discussion with YLST trial practitioners and patient and public involvement (PPI) representatives. Theoretically, the intervention is informed by the Capability Opportunity Motivation-Behaviour (COM-B) model, which is the central behaviour system of the behaviour change wheel.^39^

#### PN intervention protocol

##### Component 1: advanced notification

Participants receive an advanced notification letter (online supplemental file 3) ~3 weeks before a scheduled telephone appointment. The notification frames the appointment as an ‘Introduction to Lung Health Checks’ telephone call. It includes a low-burden leaflet (online supplemental file 4) with basic information about the Lung Health Check (similar to that sent in the control arm, but with instructions to call the team to book a risk-assessment removed) and an A5 flyer with dissent information.

##### Component 2: protocolised ‘Introduction to Lung Health Checks’ telephone call

At the prearranged time, a pathway navigator telephones the participant to introduce and discuss the lung screening offer. If contact is unsuccessful, the pathway navigator attempts two more telephone calls at different times on varied days.

**Figure 2:**
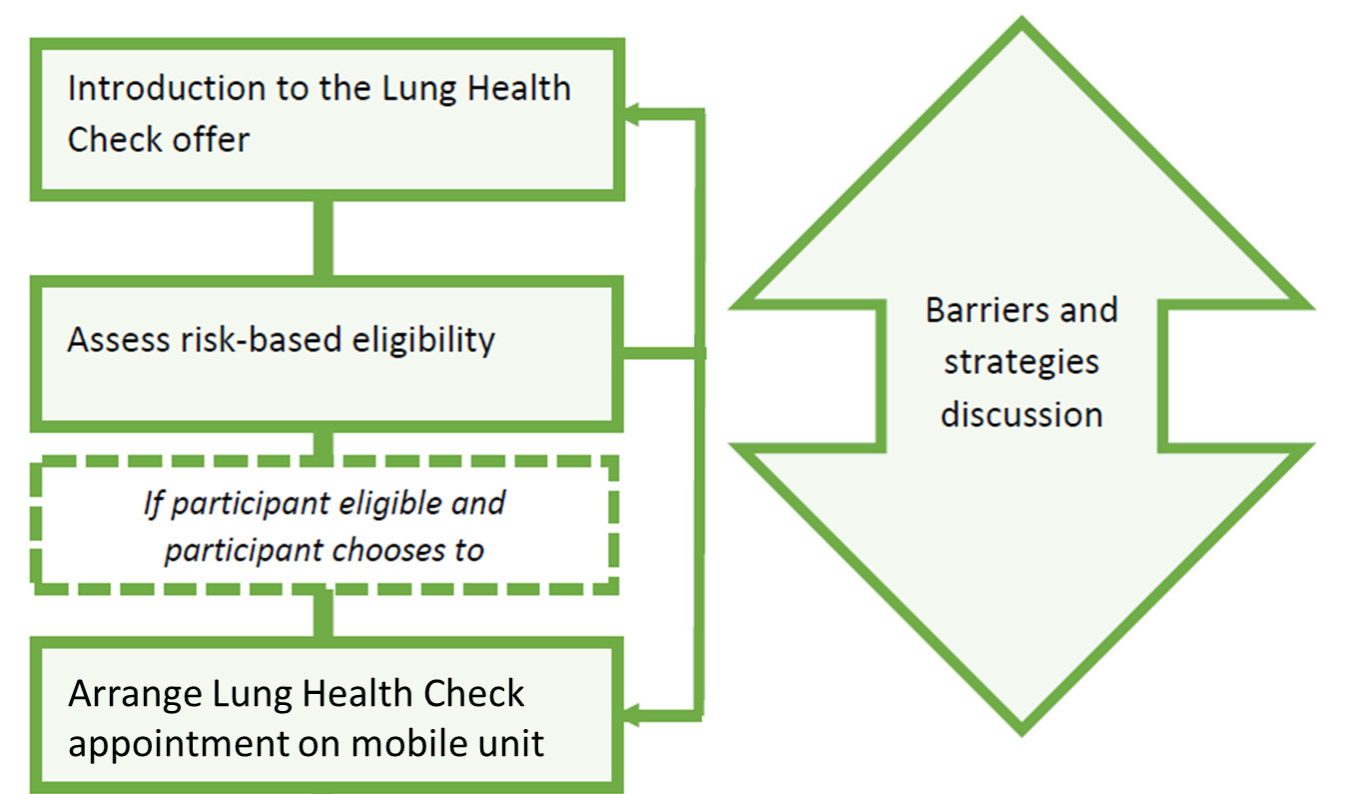
Phased pathway navigation ‘Introduction to NHS Lung Health Checks’ telephone appointment. NHS, National Health Service.

The PN team (pathway navigators) comprise trained senior clinical trials assistants, administrative officers and research nurses. Figure 2 shows the phased telephone approach as protocolised in the PN training manual (online supplemental file 5). There are four key stages, with step 3 (strategies and barriers discussion) taking place flexibly throughout the call:

1. Introducing the offer of lung screening (Lung Health Check).
2. Assessing eligibility for LDCT lung cancer screening.
3. Eliciting and addressing any barriers to capability, opportunity and motivation for attending screening using core communication competencies, problem-solving, behaviour change techniques and practical arrangements.
4. If eligible, arranging either a Lung Health Check appointment, another navigation telephone conversation (to offer further support/deliver the intended strategy as per protocol), or respecting the individual’s choice not to engage.

##### Component 3: reminder telephone calls

If the participant books a screening appointment, they will also receive reminder calls approximately 1 week and 1 day before the booked appointment.

### PN intervention training

All pathway navigators receive the protocolised training manual (online supplemental file 5). This covers the structure of the telephone call; principles of informed choice and types of non-responder (informed, misinformed, disengaged, inclined); common barriers and suggested solutions for navigators to offer for individuals to overcome them; and behavioural science-informed communication techniques (motivational interviewing, use of simple language, implementation intentions and teach-back). All pathway navigators attend a training course in motivational interviewing,^40^ and a 1-day interactive session led by behavioural scientists on the contents of the training manual before delivering the intervention. Check-ins are arranged 1 month after beginning the calls, and then every 3–6 months for feedback on experiences and to exchange knowledge.

### Control arm (usual care)

The control arm will receive the same invitation materials used to reinvite non-responders 2 years after the initial screening round of YLST (a GP-endorsed invitation letter, a low-burden reinvitation information leaflet, a dissent information statement and a follow-up reminder letter if they do not respond to the initial reinvitation).^18^ People without an available telephone number (who are therefore excluded from the PN arm) would be reinvited using the same method but not included in the analysis for this substudy, as there may be systematic bias in the characteristics of those who do not have a telephone number recorded. The letters invite the individual to telephone the YLST team to undergo a telephone-based risk assessment for a Lung Health Check appointment where LDCT lung cancer screening is offered.

## Process evaluation

### Quantitative

#### Strategies and barriers observations

At the end of each call, PN intervention acceptability will be assessed by asking individuals if they minded being telephoned and whether they found the telephone conversation helpful. After each call, the navigator will record the barriers raised, and strategies used on a standardised proforma (online supplemental file 2), informed by the COM-B framework, PPI and stakeholder review, and previous research examining barriers to lung cancer screening in low participation groups at high risk of lung cancer.^1535^,^38^ The same proforma will be completed following the completion of each telephone conversation with individuals in the control arm.

#### Fidelity observations

A subset of 200 PN calls will be observed live by a researcher who will score fidelity to the telephone protocol using a standardised proforma (online supplemental file 2). The proforma is based on similar work being conducted as part of the Yorkshire Enhanced Stop Smoking (YESS) smoking cessation trial,^41^ and guidelines on fidelity measure development.^32^ It will capture anonymous data relating to the degree to which navigators deliver the call in line with the manualised intervention. The observations will be conducted across different days with different team members delivering the calls to ensure variability in the sample. To pilot the process, the first 10 calls will be scored for fidelity by two researchers. Scores will be compared, discussed and scoring guidance adapted to ensure clarity if needed. The process will repeated for the next 10 calls to finalise scoring guidance. Inter-rater agreement will be reported for both pilot sessions to indicate reliability of the measure.

### Qualitative

#### Semi-structured interviews

At the end of each PN call, individuals will be asked if they are willing to be contacted about taking part in an interview study. Potential participants will be invited to take part by letter (posted and/or handed out during their LDCT screening appointment), with instructions to complete and return an expression of interest form by telephone, freepost letter or email to the behavioural science research team if they wish to take part. Eligible participants who are recruited in line with the purposive sampling strategy will give audiorecorded verbal consent to take part using the consent form. Semistructured interviews of ~1 hour will be carried out by a behavioural science researcher either by telephone or video call within 3 months of the PN intervention. The interview schedule (online supplemental file 6) was informed by the COM-B framework and previous research into the factors affecting lung cancer screening participation.^1535^,^38^ Open questions, prompts and probes will be used to explore the acceptability and experience of the scheduled telephone appointment approach and the types of barriers and strategies discussed.

#### Field notes on fidelity form

During fidelity observations, researchers will record field notes on contextual information related to fidelity that may be important for results interpretation (eg, navigators’ response to indirect behavioural indicators).

## Data analysis

A statistical analysis plan will be drafted by the trial statistician and reviewed by an independent statistician. Baseline characteristics by the trial arm and a diagram depicting the number and flow of patients through the trial will be presented.

### Primary analysis for the nested RCT

Descriptive analyses will be used to present numbers and proportions of interest as listed in the coprimary and secondary outcomes. To account for the related nature of the coprimary outcomes, proportions and absolute differences in proportions will be presented with associated 97.5% CIs. Formal primary comparisons of differences between groups will be made using logistic regression and adjusting for centre effects by household as appropriate.

All other statistical tests will be two sided using a 5% significance level unless otherwise specified; 95% CIs will be reported as appropriate.

### Process evaluation

#### Quantitative data

Descriptive analyses will be used to anonymously report strategies and barriers to participation; and fidelity scores to describe and quantify the extent to which the call is delivered in line with the intervention manual.

#### Qualitative data

Applied thematic analysis will be used to inductively code qualitative data from the semi-structured interviews and fidelity field notes and to interpret themes within a skeletal conceptual framework, using qualitative data analysis software NVivo.^42^ The framework will initially be based on the COM-B model but may be iteratively expanded and adapted. Initial coding will be carried out by one researcher and a subset of randomly selected transcripts coded independently by a second researcher. There will be multiple opportunities for discussion and iteration of the emerging coding framework.

#### Triangulation of mixed-methods data

A concurrent triangulation design will be followed, with quantitative and qualitative data collected and analysed separately, but converged during interpretation to inform a logic model for PN in the lung cancer screening context.

### Costs

A health economic analysis of the PN intervention will be conducted as part of the broader YLST trial aim of determining the cost-effectiveness of the YLST screening programme.^18^

## Ethics and dissemination

### REC and other regulatory review

The YLST was approved by the Greater Manchester West Research Ethics Committee (REC) (18-NW-0012) and the Health Research Authority following review by the Confidentiality Advisory Group (CAG). The PN substudy was approved by the REC and CAG as a Substantial Amendment on 5 October 2022.

### Trial monitoring and governance

Trial governance is overseen by the trial management group, the independent data monitoring committee and an independent trials steering committee. Full details are provided in the YLST protocol.^18^

### Informed consent

The YLST and nested PN study follow a service demonstration design to provide a realistic indication of uptake following PN in a real-world clinical context, improve ecological validity and avoid the Hawthorne effect,^43^ following a precedent set by earlier lung cancer screening trials.^23^ This means participants will be unaware of the research nature of the PN intervention or YLST until they attend for a Lung Health Check screening appointment, where fully informed consent will be collected to participate in the YLST. Individual consent will, therefore, not be sought before randomisation to the PN study. However, the opportunity and mechanism for dissent will be clearly described in the advance notification letter sent with details of the scheduled telephone appointment so individuals can opt-out if they do not agree to the use of this information. The opportunity to dissent from the wider YLST was also provided with all previous invitations. This approach was REC and CAG approved, and deemed acceptable by PPI representatives.

### Patient and public involvement

A PPI consultation exercise was undertaken with 12 individuals (5 male/7 female) aged between 50 and 80 years, including individuals with a smoking history who had lived experience of cancer, including lung cancer (n=7), as well as those without (n=5), to gain insight from a broad range of perspectives. The feedback was used to inform the development of the PN intervention, data collection tools and to understand the acceptability of the observational methods used for the process evaluation. All 12 individuals were supportive of the research.

### Dissemination

Study findings will be submitted for publication to relevant peer-reviewed journals in accordance with the Consolidated Standards of Reporting Trials (CONSORT) Statement^44^ and presented at conferences. A summary of the results will be provided for participants on the study website.

## Discussion

This study is the first in the UK to test whether a novel PN intervention increases uptake of lung cancer screening (Lung Health Checks) among high-risk individuals who have not responded to prior invitations within the YLST. The study will also assess the cost-effectiveness of implementing such an intervention. The mixed-methods process evaluation will provide insights into the barriers preventing people from taking part in screening, and the process through which a PN intervention may work, and its acceptability. If shown to be effective, the intervention could be integrated into the national lung cancer screening programme. This could facilitate participation by those at the highest risk of lung cancer who are otherwise least likely to respond to the screening offer (ie, people who smoke and those experiencing socioeconomic deprivation) to maximise its equity and effectiveness in reducing lung cancer deaths.

## supplementary material

10.1136/bmjopen-2024-084577 online supplemental file 1

10.1136/bmjopen-2024-084577 online supplemental file 2

10.1136/bmjopen-2024-084577 online supplemental file 3

10.1136/bmjopen-2024-084577 online supplemental file 4

10.1136/bmjopen-2024-084577 online supplemental file 5

10.1136/bmjopen-2024-084577 online supplemental file 6

## References

[1] Cancer Research UK Lung cancer Statistics 2023. https://www.cancerresearchuk.org/health-professional/cancer-statistics/statistics-by-cancer-type/lung-cancer.

[2] Solutions for Public Health (2022). Targeted screening for lung cancer in individuals at increased risk: external review against programme appraisal criteria for the UK national screening committee.

[3] Peake MD (2015). Deprivation, distance and death in lung cancer. Thorax.

[4] Mihor A, Tomsic S, Zagar T (2020). Socioeconomic inequalities in cancer incidence in Europe: A comprehensive review of population-based Epidemiological studies. Radiol Oncol.

[5] Paci E, Puliti D, Lopes Pegna A (2017). Mortality, survival and incidence rates in the ITALUNG randomised lung cancer screening trial. Thorax.

[6] Gohagan JK, Marcus PM, Fagerstrom RM (2005). Final results of the lung screening study, a randomized feasibility study of spiral CT versus chest X-ray screening for lung cancer. *Lung Cancer*.

[7] Becker N, Motsch E, Trotter A (2020). Lung cancer mortality reduction by LDCT screening-results from the randomized German LUSI trial. Int J Cancer.

[8] Pastorino U, Silva M, Sestini S (2019). Prolonged lung cancer screening reduced 10-year mortality in the MILD trial: new confirmation of lung cancer screening efficacy. *Annals of Oncology*.

[9] de Koning HJ, van der Aalst CM, de Jong PA (2020). Reduced lung-cancer mortality with volume CT screening in a randomized trial. *N Engl J Med*.

[10] The National Lung Screening Trial Research Team (2011). Reduced lung-cancer mortality with low-dose computed Tomographic screening. N Engl J Med.

[11] Field JK, Vulkan D, Davies MPA (2021). Lung cancer mortality reduction by LDCT screening: UKLS randomised trial results and international meta-analysis. *The Lancet Regional Health - Europe*.

[12] Ali N, Lifford KJ, Carter B (2015). Barriers to uptake among high-risk individuals declining participation in lung cancer screening: a mixed methods analysis of the UK lung cancer screening (UKLS) trial. BMJ Open.

[13] Schütte S, Dietrich D, Montet X (2018). Participation in lung cancer screening programs: are there gender and social differences? A systematic review. Public Health Rev.

[14] Raju S, Khawaja A, Han X (2020). Lung cancer screening: characteristics of Nonparticipants and potential screening barriers. Clin Lung Cancer.

[15] Quaife SL, Marlow LAV, McEwen A (2017). Attitudes towards lung cancer screening in socioeconomically deprived and heavy smoking communities: informing screening communication. Health Expect.

[16] Dickson JL, Hall H, Horst C (2023). Uptake of invitations to a lung health check offering low-dose CT lung cancer screening among an ethnically and socioeconomically diverse population at risk of lung cancer in the UK (SUMMIT): a prospective, longitudinal cohort study. Lancet Public Health.

[17] Crosbie PAJ, Gabe R, Simmonds I (2022). Participation in community-based lung cancer screening: the Yorkshire lung screening trial. Eur Respir J.

[18] Crosbie PA, Gabe R, Simmonds I (2020). Yorkshire lung screening trial (YLST): protocol for a randomised controlled trial to evaluate invitation to community-based low-dose CT screening for lung cancer versus usual care in a targeted population at risk. BMJ Open.

[19] Cole SR, Young GP, Byrne D (2002). Participation in screening for colorectal cancer based on a Faecal occult blood test is improved by endorsement by the primary care practitioner. J Med Screen.

[20] Hewitson P, Ward AM, Heneghan C (2011). Primary care endorsement letter and a patient leaflet to improve participation in colorectal cancer screening: results of a factorial randomised trial. Br J Cancer.

[21] Libby G, Bray J, Champion J (2011). Pre-notification increases uptake of colorectal cancer screening in all demographic groups: a randomized controlled trial. J Med Screen.

[22] Sabatino SA, Lawrence B, Elder R (2012). Effectiveness of interventions to increase screening for breast, Cervical, and colorectal cancers: nine updated systematic reviews for the guide to community preventive services. Am J Prev Med.

[23] Quaife SL, Ruparel M, Dickson JL (2020). Lung screen uptake trial (LSUT): randomized controlled clinical trial testing targeted invitation materials. Am J Respir Crit Care Med.

[24] Bartlett EC, Kemp SV, Ridge CA (2020). Baseline results of the West London lung cancer screening pilot study - impact of mobile scanners and dual risk model utilisation. *Lung Cancer*.

[25] Crosbie PA, Balata H, Evison M (2019). Implementing lung cancer screening: baseline results from a community-based ‘lung health check. *Thorax*.

[26] Percac-Lima S, Ashburner JM, Rigotti NA (2018). Patient navigation for lung cancer screening among current Smokers in community health centers a randomized controlled trial. Cancer Med.

[27] Shusted CS, Barta JA, Lake M (2019). The case for patient navigation in lung cancer screening in vulnerable populations: A systematic review. Popul Health Manag.

[28] Rivera MP, Katki HA, Tanner NT (2020). Addressing disparities in lung cancer screening eligibility and Healthcare access. An Official American Thoracic Society Statement Am J Respir Crit Care Med.

[29] McGregor LM, Skrobanski H, Ritchie M (2019). Using specialist screening practitioners (Ssps) to increase uptake of bowel scope (flexible Sigmoidoscopy) screening: results of a feasibility single-stage phase II randomised trial. BMJ Open.

[30] Richards M Report of THE INDEPENDENT REVIEW OF ADULT SCREENING PROGRAMMES in England. https://www.england.nhs.uk/wp-content/uploads/2019/02/report-of-the-independent-review-of-adult-screening-programme-in-england.pdf.

[31] Chan A-W, Tetzlaff JM, Altman DG (2013). SPIRIT 2013 statement: defining standard protocol items for clinical trials. Ann Intern Med.

[32] Walton H, Spector A, Williamson M (2020). Developing quality fidelity and engagement measures for complex health interventions. Br J Health Psychol.

[33] O’Cathain A, Croot L, Sworn K (2019). Taxonomy of approaches to developing interventions to improve health: a systematic methods overview. Pilot Feasibility Stud.

[34] McGregor L, Von Wagner C (2017). Patient navigation: bowel scope screening training manual.

[35] Cavers D, Nelson M, Rostron J (2022). Understanding patient barriers and Facilitators to uptake of lung screening using low dose computed tomography: a mixed methods Scoping review of the current literature. Respir Res.

[36] Percac-Lima S, Ashburner JM, Atlas SJ (2019). Barriers to and interest in lung cancer screening among Latino and non-Latino current and former Smokers. J Immigr Minor Health.

[37] Lowenstein M, Vijayaraghavan M, Burke NJ (2019). Real-world lung cancer screening decision-making: barriers and Facilitators. Lung Cancer.

[38] Quaife SL, Vrinten C, Ruparel M (2018). Smokers’ interest in a lung cancer screening programme: a national survey in England. BMC Cancer.

[39] Michie S, van Stralen MM, West R (2011). The behaviour change wheel: A new method for Characterising and designing behaviour change interventions. *Implementation Sci*.

[40] Rollnick S, Butler CC, Kinnersley P (2010). Motivational interviewing. BMJ.

[41] Murray RL, Brain K, Britton J (2020). Yorkshire enhanced stop smoking (YESS) study: a protocol for a randomised controlled trial to evaluate the effect of adding a Personalised smoking cessation intervention to a lung cancer screening programme. BMJ Open.

[42] Nvivo QSR International. https://www.qsrinternational.com/nvivo-qualitative-data-analysis-software/home.

[43] McCambridge J, Witton J, Elbourne DR (2014). Systematic review of the Hawthorne effect: new concepts are needed to study research participation effects. J Clin Epidemiol.

[44] Schulz KF, Altman DG, Moher D (2010). CONSORT 2010 statement: updated guidelines for reporting parallel group randomised trials. BMJ.

