# Supplementary material for: Yorkshire Lung Screening Trial (YLST) pathway navigation study: a protocol for a nested randomised controlled trial to evaluate the effect of a pathway navigation intervention on lung cancer screening uptake
Source: BMJ Open. 2024 Jul 9;14(7):e084577. doi: 10.1136/bmjopen-2024-084577 (PMC11243133; doi:10.1136/bmjopen-2024-084577)
Supplement: online supplemental file 2 [file bmjopen-14-7-s002.pdf]

Yorkshire Lung Screening Trial Pathway Navigation Study *BMJ Open* Supplementary File 2

Intervention fidelity form v0.1 02 May 2023

**Call ID**	
**Group**	☐ Never responder<br>☐ Disengaged responder
**ID of Pathway Navigator**	
**Name of observer (Researcher)**	
**Date of observation (dd/mm/yy – date recorded)**	/ /
**Call recipient gender**	
**Duration of Pathway Navigation phone call**	**Start time:**<br>**End time:**
**Stage at which Pathway Navigation phone call ended**	☐ Introduction to LHC offer<br>☐ Eligibility assessment<br>☐ Arrangement of appointment<br>☐ Strategies and barriers discussion<br>☐ Other (please describe in Notes)
**Who ended the Pathway Navigation phone call**	☐ Pathway Navigator<br>☐ Call recipient<br>☐ Mutual
**Nature of Pathway Navigation phone call**	☐ First contact/primary appointment<br>☐ Planned follow-up call<br>☐ Reminder call

Domain	Stage of phone-call	Key steps/questions for each stage	Notes
Specific activities: activities as specified by the YLST Pathway Navigation Intervention Manual	Introduction to the Lung Health Check Offer	☐ Checks identity of the person who answers the phone<br>☐ Explains who they are (name), where calling from and purpose of call<br>☐ Checks they received the appointment notification letter and leaflet<br>☐ Checks if convenient time to speak<br>☐ Checks what they already know about LHCs<br>☐ Explains what a LHC is, who they are for and what they involve<br>☐ Asks if they have any questions or worries about information given so far<br>☐ Asks if willing to have the risk-based eligibility assessment (are they willing to answer some questions about their breathing and other aspects of their lung health to see if they could benefit from having a Lung Health Check?)	
		☐ Benefits and risks discussion (optional)	
	Risk-based eligibility assessment	*Was the assessment conducted?*<br>☐ Conducted assessment when participant had confirmed they were willing<br>☐ Conducted assessment when participant had not confirmed they were willing<br>☐ Did not conduct assessment as participant was not willing<br>☐ Did not conduct assessment despite participant appearing to be willing	
		Which type of assessment was completed?<br>☐ Full assessment completed<br>☐ Full assessment partially completed but not finished<br>☐ Exclusion criteria assessment-only completed<br>☐ Exclusion criteria assessment-only partially completed but not finished	
		Was the participant eligible for a LHC appointment?<br>☐ Eligible<br>☐ Not eligible	

	Arrangement of Lung Health Check appointment	*Was an appointment offered?*<br>☐ Yes, as eligible<br>☐ Yes, even though not eligible<br>☐ No, even though eligible<br>☐ No, because not eligible	
		Did the PN emphasise the flexibility of the appointment?<br>☐ Yes<br>☐ No<br>☐ Unclear	
		Did the PN prompt the individual to add the appointment to their diary?<br>☐ Yes<br>☐ No<br>☐ Unclear	
		Was an appointment booked?<br>☐ Yes<br>☐ No	
	Strategies and barriers conversation	*When were barriers and strategies discussed: (tick all that apply)?*<br>☐ Throughout the call<br>☐ During Intro to LHC<br>☐ During risk-based eligibility assessment<br>☐ During arrangement of LHC appointment<br>☐ Distinct conversation after the other three stages<br>☐ No discussion of strategies and barriers<br>☐ Other (please describe in Notes)	
		How were the barrier and strategy discussions initiated (tick all that apply)?<br>☐ Responding to directly raised issue/barrier<br>☐ Picked up on cues<br>☐ Asked directly about barriers/worries	

-	Which communication techniques were used by the Pathway Navigator during the call?	*Which **motivational interviewing** techniques were used (tick all that apply)?*<br>☐ Open questions<br>☐ Affirmation (of strengths, efforts, past successes)<br>☐ Reflections (reflecting back to show listening + empathy)<br>☐ Summarising (ensures shared understanding, reinforcement)<br>☐ Change talk favoured over sustain talk<br>☐ Not used at all	
		*How often was **simple language** used?*<br>☐ Used exclusively<br>☐ Used mostly<br>☐ Used rarely<br>☐ Not used at all	*Examples of simple language phrases used by PN*<br>*Examples of complex phrases used by PN:*
		How often was **teachback** used?<br>☐ Used more than once<br>☐ Used once<br>☐ Not used at all	
		*How were **implementation intentions** used (tick all that apply)?*<br>☐ Used to plan travel to appointment<br>☐ Used to plan re-arrangement of conflicting responsibilities<br>☐ Used for other purpose (describe purpose in notes)<br>☐ Not used	
Other	Follow-up calls	*Was a follow-up call offered?*<br>☐ Yes<br>☐ No<br><br>*Was a follow-up call arranged?*<br>☐ Yes<br>☐ No	

	Research interview	Was the individual asked if they are willing to take part in a research interview?<br>☐ Yes<br>☐ No	
	Notes:<br>Please consider noting anything else you consider may be important for results interpretation or contextual information of the study:<br>- informed choice (informed/misinformed/disengaged/inclined)<br>- individual willingness to engage in strategies and barriers conversation<br>- PN response to indirect behavioural indicators/cues of barriers		

Strategies and barriers proforma categories

What barriers to engaging in screening were discussed?	1. Lack of awareness/knowledge about lung cancer and/or lung cancer screening<br>2. Avoidance of information about lung cancer, screening or lung cancer risk<br>3. Forgetfulness<br>4. Procrastination /failure to plan to make appointment<br>5. Competing priorities<br>6. Communication difficulties - language/health literacy<br>7. Communication difficulties - non-Native English speaker<br>8. Communication difficulties - deaf/blind disability<br>9. Travel to screening<br>10. Comorbidities or related treatments<br>11. Already undergone relevant scan so believes LHC not needed<br>12. Taking part is too much effort<br>13. Lack of social support / social network<br>14. Worried will be made to stop smoking / unwilling to stop smoking<br>15. Fear/anxiety/worry about scanning/screening procedure<br>16. Fear/anxiety/worry about having lung cancer<br>17. Fatalistic beliefs about lung cancer prognosis<br>18. Unwilling to have treatment for lung cancer<br>19. Mistrust of healthcare system<br>20. Does not feel they are at risk of lung cancer<br>21. Does not feel they could benefit from lung cancer screening<br>22. Other (please describe)<br>23. No barriers discussed
If other - please specify	Free text

What strategies to overcome the barriers to engaging in screening were discussed?	1. Explain what lung cancer is, risk factors and potential consequences<br>2. Explain purpose and potential benefits of lung cancer screening<br>3. Explained screening process step-by-step (length of LHC appointment, location, what happens, type of scan) and possible outcomes<br>4. Address lung cancer treatment concerns / misconceptions – discuss benefits of early diagnosis and treatment on lung cancer outcomes (that exist regardless of smoking status, age, current health status)<br>5. Reassurance about safety of screening process<br>6. Checked and corrected contact details (postal address / preferred phone number)<br>7. Re-arranged Introduction call to an easier time<br>8. Offered a follow-up phone call<br>9. Informed them of reminder call one week and one working day before the appointment.<br>10. Encouraged contact / provided number to call if they receive a letter that they do not understand or need to re-arrange telephone or LHC appointment<br>11. Suggested adding appointment to diary/calendar<br>12. Supported in planning travel arrangements (e.g. planned route, explain free parking at van)<br>13. Booked taxi to/from LHC appointment<br>14. Emphasised Introduction and LHC appointment flexibility (e.g. time that suits them, able to re-arrange)<br>15. Explained they can bring a friend/family member with them to support (in a carer capacity)<br>16. Held Introduction phone call over speaker phone<br>17. Problem-solve with them to help find a solution to help with responsibilities while they have the appointment<br>18. Arrange to conduct call and/or LHC appointment using LanguageLine or BSL/Deafblind interpreter<br>19. Confirm that a guide dog would be able to come to the van for the LHC<br>20. Explained the mobile vans are easily accessible, have a lift and wheelchair access, with toilet facilities nearby<br>21. Explained position on smoking (no judgements, not made to stop, but support offered to stop if they would like)<br>22. Explain self-determination (taking part is entirely their decision, no obligation, can change their mind)<br>23. Other (please describe)<br>24. No strategies discussed
If other - please specify	Free text
Is the participant willing to undergo a telephone interview about possible barriers?	Yes/No
