# Supplementary material for: Yorkshire Lung Screening Trial (YLST) pathway navigation study: a protocol for a nested randomised controlled trial to evaluate the effect of a pathway navigation intervention on lung cancer screening uptake
Source: BMJ Open. 2024 Jul 9;14(7):e084577. doi: 10.1136/bmjopen-2024-084577 (PMC11243133; doi:10.1136/bmjopen-2024-084577)
Supplement: online supplemental file 3 [file bmjopen-14-7-s003.pdf]

LEEDS LUNG HEALTH CHECK

NHS
The Leeds Teaching Hospitals
NHS Trust

Dear <<Firstname>> <<Surname>>

NHS Number: <<NHSNumber>>

Your Introduction to NHS Lung Health Checks telephone appointment

You are invited for an ‘Introduction to NHS Lung Health Checks’ telephone appointment. The calls are being organised by Leeds Teaching Hospitals NHS Trust.

Date and time of your Introduction to NHS Lung Health Checks telephone call:
<<appointment day>>, <<appointment date>> at <<appointment time>>

What is an NHS Lung Health Check?
They are for people aged 55 to 80 who smoke or used to smoke. You are invited to this Introduction telephone appointment whether or not you have any current lung problems, even if you feel fine. The appointment will take approximately 30 minutes.

More than 6,000 people in Leeds have already had an NHS Lung Health Check, supported by over 70 GP surgeries.

What you need to do now

- Take a moment to check you will be free at the scheduled telephone appointment time and add it to your calendar.
- If you do not want the appointment, or want to change the date or time, call the Lung Health Check number **0113 39 26688** Monday to Friday, 9am-4:30pm to speak to a member of our team.
- For information about what to expect, see the leaflet **M.O.T for your lungs**

Your health information

Everyone invited for a “Introduction to NHS Lung Health Check” call will have information about their health collected throughout their lifetime, even if they choose not to attend. Everyone who takes part in this “Introduction to NHS Lung Health Checks” telephone call will also have information collected about the reasons why they choose to attend or not to attend. Some calls may also be observed by members of the Leeds NHS Lung Health Check research team to understand how well the practitioner delivers the telephone call. This information will be used anonymously in a research study to help assess the effectiveness of the Lung Health Checks and help us understand how best to support future patients to be able to attend them.

If you do not want your health information to be used in this way, you can opt out. If you would like to opt out, please let us know by phoning 011339266 88. You can also e-mail leedslunghealthcheck@nhs.net or write to “Leeds Lung Health Check, Leeds Chest Clinic, Leeds General Infirmary, LS1 3EX”.
