# Supplementary material for: Yorkshire Lung Screening Trial (YLST) pathway navigation study: a protocol for a nested randomised controlled trial to evaluate the effect of a pathway navigation intervention on lung cancer screening uptake
Source: BMJ Open. 2024 Jul 9;14(7):e084577. doi: 10.1136/bmjopen-2024-084577 (PMC11243133; doi:10.1136/bmjopen-2024-084577)
Supplement: online supplemental file 4 [file bmjopen-14-7-s004.pdf]

MOT FOR YOUR LUNGS

A new **FREE NHS LUNG HEALTH CHECK** for people aged 55 to 80 in Leeds

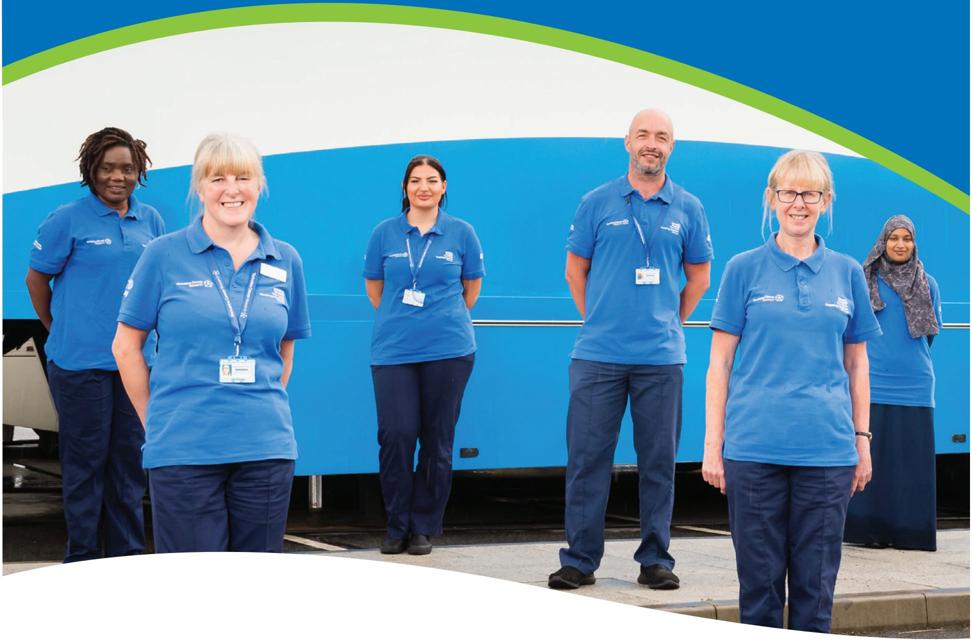

Leeds Lung Health Check

Funded by Yorkshire Cancer Research

WHAT WILL HAPPEN

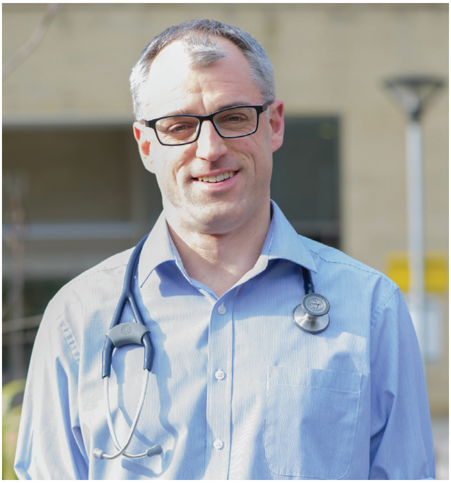

A specially trained healthcare professional will telephone you at the **appointment time in your letter**.

They will explain what a Lung Health Check is and ask some questions about your breathing and lung health to see if you could benefit from one.

They might also offer you a face-to-face appointment if they decide you could benefit. You'll have time to ask any questions. For more information or to re-arrange your appointment call:

0113 392 6688

The lung health check can spot early problems with your lungs that we may be able to treat more easily

Suzanne, Nurse
Leeds Teaching Hospitals

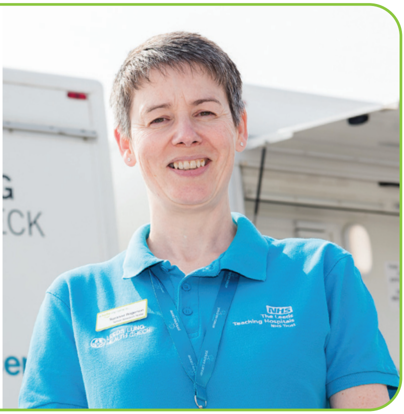

© The Leeds Teaching Hospitals NHS Trust • V1.0 PN MOT 19th July 2022 IRAS 235803
This leaflet is a locally-adapted version of a leaflet originally created by the Department of Behavioural Science and Health and Division of Medicine at University College London (UCL) in partnership with Resonant Media, which is licensed under CC BY.
Produced by: Medical Illustration Services • MID code: 20220712_001/DG

LN005362
Publication date 09/2022
Review date 09/2024
