# Supplementary material for: Yorkshire Lung Screening Trial (YLST) pathway navigation study: a protocol for a nested randomised controlled trial to evaluate the effect of a pathway navigation intervention on lung cancer screening uptake
Source: BMJ Open. 2024 Jul 9;14(7):e084577. doi: 10.1136/bmjopen-2024-084577 (PMC11243133; doi:10.1136/bmjopen-2024-084577)
Supplement: online supplemental file 5 [file bmjopen-14-7-s005.pdf]

Yorkshire Lung Screening Trial
Pathway Navigation

TRAINING MANUAL

Guiding people around obstacles to taking part in lung cancer screening as part of Leeds Lung Health Checks

Contents

SECTION 1: Overview and Core Concepts ... 3
Purpose of this manual ... 3
What is this manual? ... 3
Why have I received this manual? ... 3
How should I use this manual? ... 3
What is Pathway Navigation? ... 4
Brief history of pathway navigation ... 4
A note on terminology ...
Defining Pathway Navigation ... 5
What evidence is there that Pathway Navigation improves cancer screening uptake? ... 5
Where does Pathway Navigation fit in the YLST? ... 6
Recap of YLST aims ... 6
Why introduce Pathway Navigation into the YLST? ... 6
Overview of the YLST Pathway Navigation Study ... 7
Pathway Navigation to Screening in YLST: Step-by-Step Process ... 9
SECTION 2: Focus on the Introduction to Lung Health Checks Telephone Appointment ... 10
Purpose of the telephone appointment ... 10
Structure of the telephone appointment ... 11
The role of the Navigator ... 12
The importance of informed choice ... 13
Telephone appointment: step-by-step guide ... 14
Introducing the Lung Health Check offer ... 14
Conducting the risk-based eligibility assessment ... 17
Arranging the Lung Health Check appointment ... 18
Discussing barriers and strategies ... 19
Key techniques to use during the telephone appointment ... 27
Motivational Interviewing ... 27
Communication techniques ... 29
Receiving calls ... 32
Handling queries about the national lung cancer screening programme ... 33
SECTION 3: Activities to consolidate learning ... 35
TASK A: Using simple language ... 35
TASK B: Case study discussions ... 36
TASK C: Practice Session - Role play ... 38
References and resources ... 40

Appendix 1. Barriers and Strategies PPM+ form categories 41

SECTION 1: Overview and Core Concepts

Purpose of this manual

What is this manual?

This is your step-by-step guide to delivering the Yorkshire Lung Screening Trial Pathway Navigation telephone intervention.

It is designed to accompany your in-person training session.

It is divided into 3 core sections:

- Overview and core concepts – what is Pathway Navigation and how does it fit into the YLST?
- Activities to consolidate learning – practicing key Pathway Navigation techniques
- Tip sheet – to keep with you during the calls you make

Why have I received this manual?

You are helping to deliver the Pathway Navigation intervention as part of the third round of the Yorkshire Lung Screening Trial Lung Health Checks programme.

Before delivering the intervention, it is important to complete training so you understand what Pathway Navigation is, and how it should be delivered. This also helps make sure that you carry out the Pathway Navigation intervention in a similar way to others in the team (known as 'fidelity').

How should I use this manual?

It contains all the core information that will be covered during the in-person training.

It is yours to keep. Feel free to annotate or take notes on it to support what you learn during the in-person training.

You might find it helpful to keep this manual with you when making your Pathway Navigation telephone calls – particularly the Tip Sheet (Page 38) and Strategies and Barriers guide (Page 19).

What is Pathway Navigation?

Brief history of pathway navigation

Harold Freeman first introduced the concept of pathway navigation in the 1990s in relation to his work helping women from low-income backgrounds in New York access preventative cancer services.

Freeman recognised that the healthcare system was complex and difficult to navigate. This meant many women, often those from lower-income or minority backgrounds, were unable to access services (such as cancer diagnostic services) that could lower their risk of dying from cancer.

Freeman wanted to help guide people through these complex systems, enabling them to overcome barriers to accessing services. He termed this process Patient Navigation.

A note on terminology

Pathway Navigation is also sometimes known as Patient Navigation (the term Freeman used).

In this study, we are using the term **Pathway Navigation** as many people going through the YLST Lung Health Checks are not 'patients' – they are people who we are helping consider the offer of lung cancer screening and navigate the lung cancer screening pathway.

Figure 1 shows how barriers to navigating complex healthcare pathways can lead to **inequities in access** to healthcare services, and how Pathway Navigation can help.

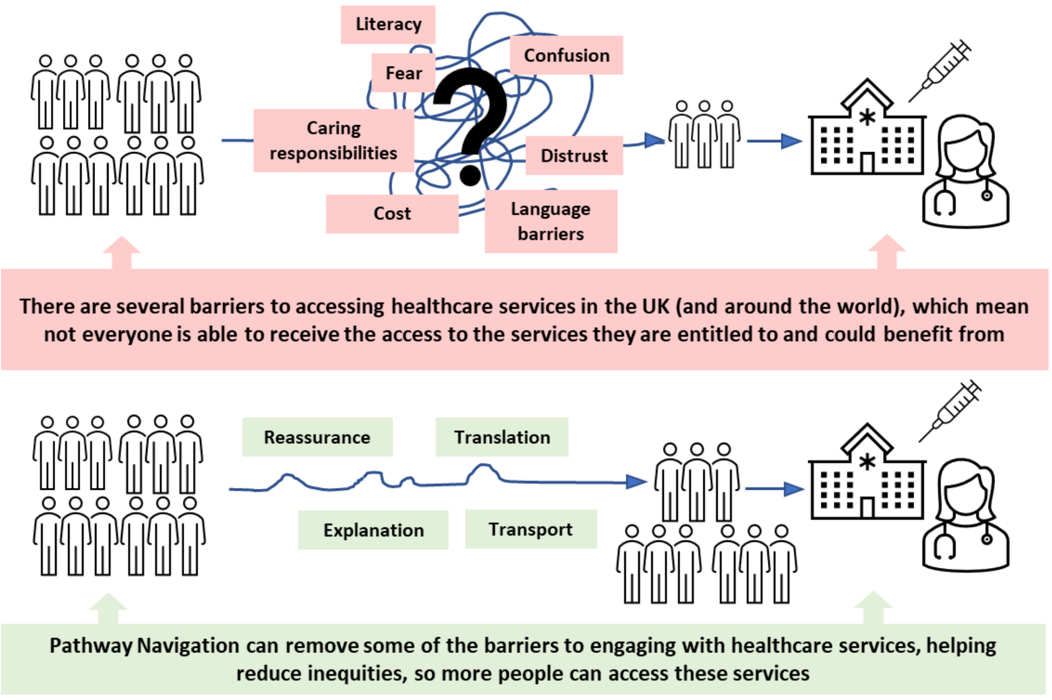

Figure 1. Pathway Navigation can help more people access healthcare services

Defining Pathway Navigation

Pathway (or Patient) Navigation can be defined as[1]:

> ***"A process by which an individual, a pathway navigator, guides people in overcoming barriers to healthcare services access to facilitate timely access to care"***

In the context of cancer care, this definition can be broken down into **3 distinct phases** of Pathway Navigation:

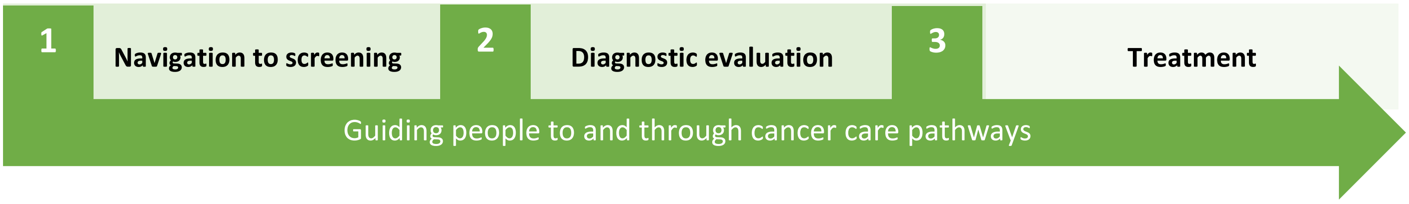

This training will focus on the first phase – **Navigation to Screening**.

What evidence is there that Pathway Navigation improves cancer screening uptake?

- **Cancer screening:** Pathway Navigation (PN) has been tested in many different types of cancer screening programme. A systematic review, which evaluated evidence from trials of pathway navigation interventions in cancer screening, found that PN improved participation in colorectal, breast and cervical cancers in populations adversely affected by health disparities[2]– see Box 1.

- **Lung cancer screening:** There is also evidence from a randomised controlled trial in the US that Pathway Navigation improves lung cancer screening participation in underserved populations. In fact, a recent American Thoracic Society statement recommended pathway navigation be integrated in lung screening programmes to reduce disparities in uptake[3].

- But, so far there aren't any studies testing Pathway Navigation for UK lung screening programmes.

Box 1. Populations adversely affected by health disparities

**Populations adversely affected by health disparities:**
Racial and ethnic minority populations<br>Socioeconomically disadvantaged populations<br>Underserved rural populations<br>Sexual and gender minority populations, and others subject to discrimination.

[1] *Definition adapted from* Peart A, Lewis V, Brown T, et al. Patient navigators facilitating access to primary care: a scoping review BMJ Open 2018;8:e019252. doi: 10.1136/bmjopen-2017-019252

[2] Nelson HD, Cantor A, Wagner J, et al. Effectiveness of Patient Navigation to Increase Cancer Screening in Populations Adversely Affected by Health Disparities: a Meta-analysis. J Gen Intern Med. 2020 Oct;35(10):3026-3035. doi: 10.1007/s11606-020-06020-9.

[3] Lung cancer screening guidelines. American Cancer Society. Accessed January 15, 2021. https://www.cancer.org/health-care-professionals/american-cancer-society-prevention-early-detection-guidelines/lung-cancer-screening-guidelines.html

Where does Pathway Navigation fit in the YLST?

Recap of YLST aims

The Yorkshire Lung Screening Trial (YLST) invites ever-smokers aged 55-80 years who are registered with a general practice in Leeds to take part in a community-based lung screening programme (Lung Health Checks).

So far there have been two rounds of invitations:

- Round 1 (November 2018 – February 2021)
- Round 2 (March 2021 – October 2022)

The primary aims of the Yorkshire Lung Screening Trial (YLST) are to:

- Measure participation rates for people invited to a community-based lung screening programme (Lung Health Checks)
- Compare the performance of different methods of lung-cancer risk assessment
- Compare the clinical outcomes (advanced lung cancer rates and lung cancer deaths) between people who receive an invitation to a Lung Health Check versus those who are not invited.

So far, YLST has demonstrated that **screening is deliverable within the real-world NHS** even during a pandemic, with **over 160 patients diagnosed with lung cancer** during the baseline round (2.4% of those screened), the vast majority at early stage and receiving treatment aiming at cure.

Why introduce Pathway Navigation into the YLST?

The Pathway Navigation intervention will be delivered to people as part of a third round of invitations taking place between November 2022 – September 2023.

- In the first round of the YLST, **only 50% of people invited took up the offer** to have a Lung Health Check.
- Uptake of Lung Health Checks was skewed towards those from more affluent areas, and towards those people who had quit smoking (compared to those who continue to smoke). This means people at the highest risk of lung cancer (and so most likely to benefit from the lung cancer screening programme) were the least likely to take part.
- It's important we find a way to engage these groups at higher risk of lung cancer and reduce inequalities in participation in Lung Health Checks.
- Evidence suggests many of those not responding to their lung cancer screening invitations do so because of **modifiable factors** which limit their ability to attend or undermine their motivation to take part. These include **emotional barriers** (e.g., fear of diagnosis) and **practical barriers** (e.g., difficulties making travel arrangements).
- In the YLST, we want to test if using Pathway Navigation to remove, or help people overcome, these barriers could improve uptake of Lung Health Checks and reduce inequalities in participation in lung cancer screening.

Overview of the YLST Pathway Navigation Study

Who will receive Pathway Navigation?

Pathway Navigation will be introduced into the YLST for two groups of people:

Repeat Non-Responders – people who have not responded to written invitations during the first two rounds of YLST

- 50% of repeat non-responders will receive the **Pathway Navigation** intervention

  (randomly allocated)

- 50% of repeat non-responders will receive **usual care** (a standard re-invitation letter; no Pathway Navigation

  (randomly allocated)

Disengaged Responders – people who completed the telephone triage in response to the first two rounds of YLST written invitations and were eligible for screening, but disengaged at some point afterwards (e.g., not booking or attending the appointment at all or only having one scan)

- All disengaged responders will receive the **Pathway Navigation** intervention

What does the Pathway Navigation intervention involve?

In simple terms the intervention is made up of the following components:

1. **A postal notification** of a pre-booked Introduction to Lung Health Checks telephone appointment
2. A 10-15 minute **Introduction to Lung Health Check telephone appointment** delivered by trained practitioners (and up to 5 follow up phone calls if the person does not answer)
3. **Provision of motivational and practical support** (e.g. overcoming negative preconceptions, arranging translators or transport) to overcome barriers discussed during the telephone appointment

Details of how these intervention components should be delivered are covered in **Section 2** of this manual.

How will the Pathway Navigation intervention be evaluated?

- Practitioners delivering the Pathway Navigation Intervention will **record the barriers and strategies** discussed during the Introduction to Lung Health Checks telephone appointment after they have completed each telephone call. This information will be used to understand the range of barriers and strategies used, as well as how commonly they occur and are used.
- Individuals will also be asked if they are willing to be contacted about taking part in an **in-depth interview** at a later date. Researchers from Queen Mary University of London will carry out these interviews to understand individuals' experiences of the Pathway Navigation intervention to better understand which aspects work, which don't, and why.
- **The effectiveness** of Pathway Navigation will be assessed by comparing the numbers of people undergoing the initial telephone eligibility assessment, the numbers found eligible for screening, and the numbers attending the Lung Health Check between repeat non-responders who receive the Pathway Navigation intervention, and those who do not. To measure this, people who have not responded to the previous two rounds of invitations will be randomly assigned to either receive the Pathway Navigation intervention or to receive usual care (another re-invitation letter). The costs of delivering pathway navigation will also be worked out to understand if it is not only effective, but affordable for national roll-out.

Pathway Navigation to Screening in YLST: Step-by-Step Process

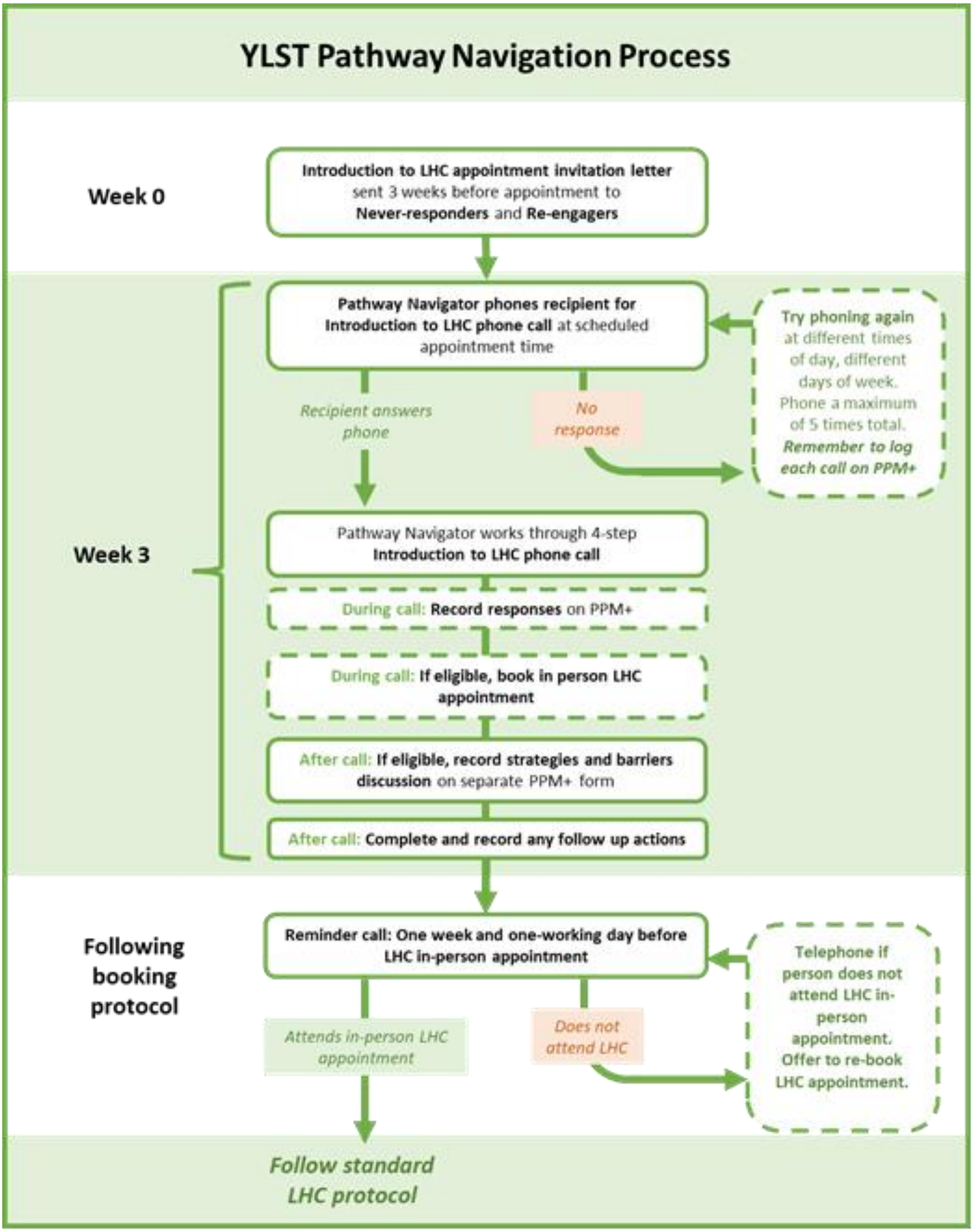

SECTION 2: Focus on the Introduction to Lung Health Checks Telephone Appointment

Purpose of the telephone appointment

The aims of the Introduction to Lung Health Checks telephone appointment are to help individuals overcome avoidable barriers to attending a Lung Health Check and provide information about the Lung Health Checks so that an individual can make an informed choice about whether or not to:

A. Undergo the telephone risk-based eligibility check → *If eligible* → B. Make a Lung Health Check appointment.

The phone-call is an opportunity to:

- Identify and discuss **any potential barriers** to an individual undergoing the eligibility check, and/or making a Lung Health Check appointment;
- Discuss **any strategies** that the individual can use to overcome those barriers, and any support the YLST programme can provide to help.
- This includes provision of information or reassurance to help overcome psychological or emotional barriers, such as fear that may be caused by difficult experiences or inaccurate beliefs (e.g., lung cancer is always fatal).

Structure of the telephone appointment

There are 4 parts of the telephone appointment.

(1) An introduction to the offer of the Lung Health Check, (2) the risk-based eligibility check, (3) arranging a Lung Health Check appointment and (4) discussion of barriers and strategies.

The **strategies and barriers conversation should happen flexibly throughout the conversation**, sometimes right from the start of the conversation.

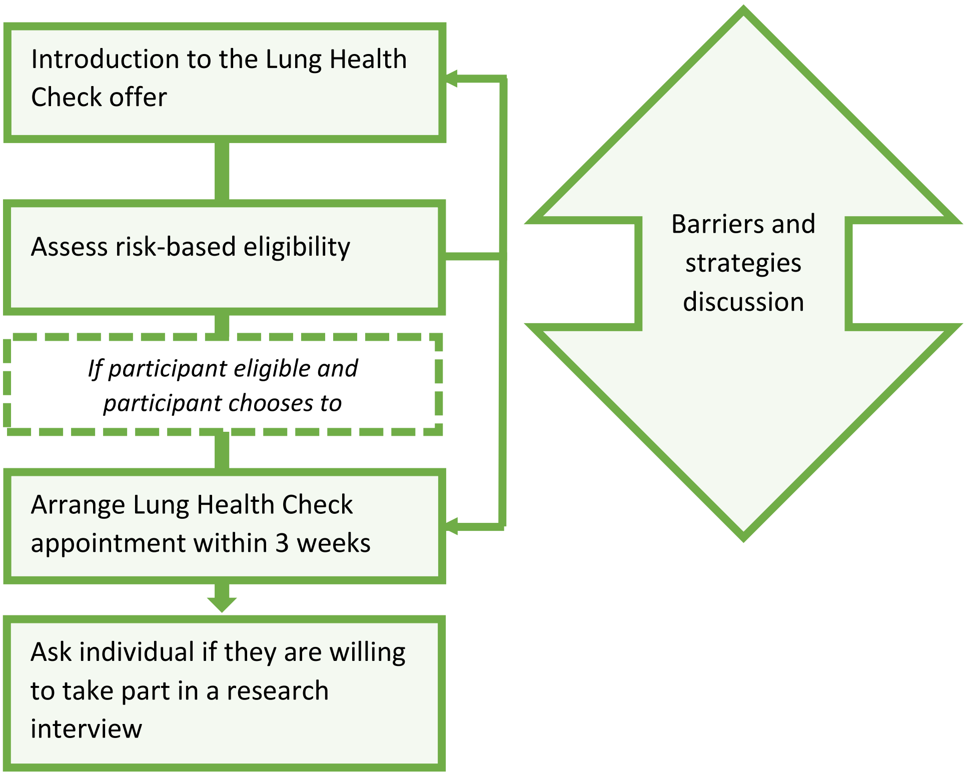

The role of the Navigator

The roles of the navigator are to:

- **Provide information** about what the Lung Health Check is, why they have been invited and why it is important, including risks and benefits
- **Conduct the risk-based eligibility check** for individuals who agree to (informed choice)
- **Identify barriers** to engaging with Lung Health Checks (the eligibility screen or the Lung Health Check at the mobile van)
- **Offer solutions** to overcome the identified barriers.
- **Record the call on PPM+** (Pathway Navigator or Re-Engager form AND Strategies/Barriers Form)
- **Ask the individual if they would be willing to be contacted about being interviewed** as part of a study to understand more about people's decision to have a Lung Health Check and their experience of the telephone appointment.
- **Receive calls from individuals** who have received an Introduction to Lung Health Checks appointment notification, or who are ringing before or after an appointment.

✓ Be positive and approachable
✓ Use plain and simple language
✓ Ask open-ended questions
✓ Listen carefully to what an individual is telling them, and problem-solve to find solutions

The role of the navigator is not to:

- **Convince somebody** who has sufficient knowledge and understanding of lung cancer and Lung Health Checks to take part against their will (see The Important of informed choice section on Page 13).
- **Become a personal point of contact** for any individual throughout the Lung Health Check programme or beyond
- **Offer personal solutions** (e.g. a lift in your own car to the mobile van) to help an individual attend the Lung Health Check programme beyond the solutions listed in the Barriers and Strategies section of this manual (see Page 19).
- **Give medical advice or offer person opinion** on health matters, beyond the appropriateness of having a Lung Health Check

✗ Do not be judgemental, negative or rude
✗ Do not make promises that cannot be kept
✗ Do not force an individual to elaborate if they are not comfortable doing so. Sometimes people are not able or comfortable to discuss things in front of others. If this is the case, you can offer to re-arrange the phone call to another time when they might feel more able to talk openly.

The importance of informed choice

- When we talk about 'improving uptake' and 'removing barriers' it is important to remember that we must not force anyone to have a Lung Health Check. It is up to an individual to decide if they want to take part, based on a clear understanding of what is involved, as well as the risks and benefits. This is called making an **informed choice about participating.**
- Research shows that sometimes people choose not to take part in screening without having received or understood all the information required to make an informed choice. For example, this can happen if emotions lead them to avoid the information, or if they have difficulties reading information that can lead to misunderstanding. It is our responsibility, so far as possible, to help ensure individuals don't make an **uninformed choice about not participating**.
- Pathway Navigation helps people achieve a clear understanding of what Lung Health Checks involve, the risks and benefits. And, if people decide based on that information that they would like to take part, helps people overcome any practical or psychological barriers to attending the screening.
- In the context of the YLST, we can think of four groups of people invited to take part in screening who do not engage or attend:

Types of non-engager/non-attender	
**Informed**	**Misinformed**
Someone who has sufficient knowledge and understanding of lung cancer and the Lung Health Check process, its risks and benefits.<br><br>It perfectly acceptable for them to continue not to want to take part and they should feel that we fully accept their choice.	Someone who has misconceptions about lung cancer or the Lung Health Check process, its risks and barriers, or has emotional barriers, which stop them engaging with or attending Lung Health Checks.<br><br>The aim of Pathway Navigation for these people is to help explain any misconceptions, correct any misinformation, and offer strategies to overcome any emotional and/or practical barriers stopping them from engaging.<br><br>Again, if after several suggestions there is no resolution, it is fine to accept their choice not to engage or attend.
**Disengaged**	**Inclined**
Someone who had limited or no awareness of lung cancer, lung cancer screening and/or Lung Health Checks.<br><br>The aim of Pathway Navigation is to give simple information about the Lung Health Check programme. And, if once armed with this information, they would be interested in taking part, helping them to overcome any emotional and/or practical barriers stopping them from engaging.	Someone who is interested in, or had intended to engage in the Lung Health Check programme, but might have forgotten to respond to their invitation or attend an appointment. This could be down to conflicting priorities, procrastination or simply forgetfulness.<br><br>The aim of Pathway Navigation is to act as a reminder that the option for screening is still available, and provide people with their own strategies to manage conflicting priorities.

Telephone appointment: step-by-step guide

Introducing the Lung Health Check offer

During the introduction, you should cover the following information.

Use the communication techniques covered on Page 27-31 to ensure the information is communicated clearly, understood and acted upon: motivational interviewing, simple language, implementation intentions, and teach-back.

**Check identity of the person who answers the phone**	If not correct, ask to talk to them. If the number is incorrect, apologise, record this on PPM+ and source correct number.
**Explain who you are, where you are calling from and the purpose of the call**	Say your name, and that you are calling from Leeds Teaching Hospital for their Introduction to Lung Health Checks telephone appointment.
**Check they received the appointment notification and leaflet**	If not, check postal address on record and correct if there is an error
**Check if now is a convenient time to speak**	The appointment will last around 10 -15 minutes.<br>If not, offer to reschedule. Record time and purpose of call-back in calls spreadsheet. Suggest participant adds time to their diary.
**Check what they already know about Lung Health Checks?**	Some people (especially people in the Disengaged Responders group) may already have a good understanding of what a Lung Health Check is. It is good to establish this at the start of the call by asking if they have heard of Lung Health Checks and what they already know about them.<br><br>This conversation may lead people to discuss barriers that led to them dis-engaging, so be ready to engage in the Barriers/Strategies conversation at this stage (see Page 19).
**Explain what a Lung Health Check is, who they are for, and what they involve**	Tailor this part of the conversation to the level of knowledge established through the previous question.<br><br>A Free NHS Lung Health Check for people aged 55 to 80 in Leeds who are current or former smokers.<br><br>Invited whether you feel fine or not, and whether or not you have any lung problems<br><br>The Lung Health Check is like an MOT for your lungs to see if they are in good working order.

The appointment will take place at a community setting (like a supermarket carpark) close to home and will take around an hour.

They are run by specially trained nurses, who can find out how well your lungs are working. At the appointment, a trained nurse will ask some questions about their health and lung health. They will be asked to do a blowing test, which measures how well their lungs work. They might also be offered a Lung CT scan, which is a type of X-ray for your lungs.

Most people will find out that their lungs are working fine. Some people may benefit from further tests or treatment either from their GP or Hospital Doctors.

Explain the risks and benefits of a Lung Health Check

Benefits of a Lung Health Check

Totally free, local and easy to get to – the check takes place in a community setting close to your home

As part of the Lung Health Check they may be offered a lung CT scan to check for any change in your lungs that might need treatment. One of the particular things we are looking for is any early signs of **lung cancer**.

Checking for cancer in this way is called screening. They may have heard of screening for other types of cancer such as breast cancer or bowel cancer. For all types of cancer screening, there are benefits but also some downsides. When they come for their Lung Health Check, we will explain these so that they can make up their own mind and decide for themself if they would like a scan.

Benefits of lung cancer screening
When lung cancer is found through screening it is usually very small and only in the lungs. This means treatment can cure most patients. When lung cancer is found by screening and is successfully treated, people live an average of ten years longer than people who have cancer found in other ways.

Are there any risks from a CT scan?
CT scanners use a small amount of radiation to produce pictures of your lungs. Exposure to radiation can itself cause problems (very rarely actually causing cancer). By using very modern CT scanners we can reduce the amount of radiation needed. Our scanner uses levels of radiation that are about the same as those found in the environment over the past year. The chance of the scan saving your life by finding an early cancer is much greater than the risk of the scan causing you any harm.

**Ask if they have any specific questions or worries about the information they have been given so far**	Refer to the Barriers and Strategies section as required to handle any particular concerns that are raised (see Page 19)
**Ask if they are willing to answer some questions about their breathing and other aspects of their lung health to see if they could benefit from having a Lung Health Check**	If not, explore reasons why not and identify any barriers/strategies (see Page 19).
**Move on to risk-based eligibility assessment**	If yes.<br>If no, ask if they would be willing to take part in a semi-structured interview

Conducting the risk-based eligibility assessment

If the individual is happy to check their eligibility for the Lung Health Check, follow the question prompts on the PPM+ form to work through the eligibility check. Record answers on the appropriate form during the call.

Repeat Non-Responders *have to complete the full risk-based eligibility assessment. Disengaged Responders only have to answer the questions relating to specific exclusion criteria, as they have already previously completed the eligibility check.*

Question	Who asked
**When was the last time you smoked a cigarette?**	Repeat Non-Responders only
**Any other comment**	Repeat Non-Responders only
**Height - measurement type**	Repeat Non-Responders only
**Height - measurement units**	Repeat Non-Responders only
**Height - current height**	Repeat Non-Responders only
**Weight - measurement type**	Repeat Non-Responders only
**Weight - measurement units**	Repeat Non-Responders only
**Weight - current height**	Repeat Non-Responders only
**Weight - are there clinical factors affecting patient's weight**	Repeat Non-Responders only
**Previous respiratory diagnosis**	Repeat Non-Responders only
**Personal history of previous cancer**	Repeat Non-Responders only
**Family history of lung cancer (if not known put no)**	Repeat Non-Responders only
**If family history, was it first degree relative (father, mother, brother, sister, son, daughter)**	Repeat Non-Responders only
**How many first degree relatives were affected**	Repeat Non-Responders only
**Age of youngest first degree relative at time of diagnosis of cancer**	Repeat Non-Responders only
**Job or activity with asbestos exposure**	Repeat Non-Responders only
**If ever smoked cigarettes, age when started?**	Repeat Non-Responders only
**If previously smoked cigarettes but now quit, age when quit?**	Repeat Non-Responders only
**Average number of cigarettes per day whilst smoking**	Repeat Non-Responders only
**Average grams of tobacco smoked per week if roll-ups whilst smoking**	Repeat Non-Responders only
**What was your highest level of education?**	Repeat Non-Responders only
**How would you describe your ethnicity?**	Repeat Non-Responders only
**Exclusion criteria**	
**Have you had a CT scan of your chest in the last 12 months?**	Repeat Non-Responders AND Disengaged Responders
**Do you have an active diagnosis of cancer?**	Repeat Non-Responders AND Disengaged Responders
**Have you had a lung cancer diagnosis in the last 5 years?**	Repeat Non-Responders AND Disengaged Responders
**Is there another exclusion criteria?**	Repeat Non-Responders AND Disengaged Responders
**Other exclusion criteria details**	Repeat Non-Responders AND Disengaged Responders

Arranging the Lung Health Check appointment

If the eligibility check shows that the individual is eligible for the Lung Health Check, you should now offer to arrange a Lung Health Check appointment at the mobile van.

If the offer is accepted, book the appointment in PAS as per standard procedure.

***Remember to**:*

Emphasise the flexibility of the appointment – encourage them to make it now, but if it needs to be changed, they can always ring back to re-arrange. Provide the number for them to do this.

Prompt them to add the appointment to their diary/phone whilst on the call and ask them how they will travel so they plan out their journey (see Implementation Intentions on Page 30)

Discussing barriers and strategies

The barriers and strategies discussion is part of the Pathway Navigation appointment that should be used flexibly throughout the conversation.

Use your judgement to gauge when would be a good time to explore any particular concerns an individual might have about undergoing the risk-based eligibility assessment, or attending the Lung Health Check, based on how the individual is engaging in the conversation from the outset.

You can respond directly to issues they raise themselves:

I'd like to, but I won't be able to get there…

There's no point, I'm not going to give up smoking

You can pick up on cues they give you indirectly, such as:

- Lack of clear understanding when using the teach-back method (see Page 31)
- Closed off/unwilling to participate openly in conversation

And you can ask directly:

- *Is anything in particular worrying you about attending the Lung Health Check?*
- *Can I ask what is stopping you from wanting to find out if you are eligible?*
- *Can you think of any support you might need from us that would make it easier for you to be able to attend the appointment?*

On the next page is a summary list of possible barriers an individual might raise, and strategies you can use as a Navigator to help them overcome those barriers. If an individual raises a barrier not on the list, use your problem-solving and motivational interviewing skills to think of possible strategies that an individual could use to overcome the issue they have raised.

Remember informed choice
When offering solutions or strategies, this is your chance to put the individual in the best possible position to be able to make an informed choice about whether or not to engage. After receiving and understanding all the information, and hearing all of the strategies available to support them to attend, if an individual decides not to undergo the eligibility check, or book a Lung Health Check appointment, the individual must feel that we fully accept their choice.

Possible barriers and strategies you can use

At the end of the phone call, after the individual has hung up the phone, remember to open the separate Barriers and Strategies form on PPM+ and record all Barriers discussed and Strategies used during the phone call. Select as many as is appropriate. See Appendix 1 for a copy of the categories on the form.

Barrier category	Specific barrier	Possible strategy/solution[1]
This is the Barrier category you should record on the PPM+ form		NB/ All solutions should be offered whilst continuing to emphasise it is completely their choice whether they have the LHC, and validating the concerns/barriers raised as important
	Awareness / knowledge	
**Lack of awareness/knowledge about lung cancer and/or lung cancer screening**	About lung cancer:<br>• What lung cancer is<br>• Lung cancer risk factors<br>• Potential consequences of lung cancer	• Explain what lung cancer is, risk factors and potential consequences<br>• Discuss benefits of early diagnosis (that exist regardless of smoking status, age, current health status)
	About lung cancer screening:<br>• Didn't receive/doesn't remember receiving previous invitations<br>• Doesn't know what lung cancer screening is<br>• Doesn't know potential benefits of lung cancer screening	• Explain what lung cancer screening is, its purpose and potential benefits<br>• **If did not receive previous invitations:** Apologise and check address is correct<br>• **If LHC appointment arranged:** Inform them there will be a reminder call one week and one working day before the appointment.<br>• Explain that if they receive a letter and do not understand, they can call the number [give number: 0113 392 6688]
**Avoidance of information about lung cancer, screening or lung cancer risk (avoiding negative emotions)**	• Don't want to receive information about lung cancer (or other cancer)<br>• Don't want to know about their personal risk of lung cancer<br>• Don't want to know if they have lung cancer	• Explore reasons for why they do not want to know (e.g. Are they scared of the outcome? Hold self-stigmatising views? Afraid of having to stop smoking? Treatment concerns?). The underlying reason may link to another solution/action we can offer.<br>• Explain that they do not need to be told their personal risk of lung cancer – only whether or not they are at increased risk meaning they meet the threshold for screening eligibility and could benefit from a scan

		• Offer a call back in a few days' time if the person wants time to think/is in an emotional state
Personal capability		
**Forgetfulness**	Forgot to book or attend following previous invitations	• **If eligible:** Offer to book LHC on call today<br>• **If LHC appointment arranged:** Inform them there will be a reminder call one week and one working day before the appointment<br>• **If LHC appointment arranged:**<br>○ Suggest adding appointment to diary/calendar whilst on the phone.<br>○ Support with making an attendance plan (e.g., travel arrangements)?
**Procrastination/failure to plan to make an appointment**	Procrastination/intended to but did not get around to making an appointment	
**Competing priorities**	• Unable to or worried about taking time off work<br>• Caring responsibilities (partner, childcare, pets, other)<br>• Clashed with cultural/faith festival<br>• Already taking part in other research<br>• Other hospital appointments	• Emphasise flexibility of scheduling appointment – we can make appointment at a time that suits them (8am – 5pm Monday-Saturday), we can provide letter for workplace, we can make a provisional appointment that they can change<br>• Explain how long the process will take<br>• Remind them of the number they can call if they need to cancel/re-arrange<br>• Explain they are able to bring a friend/family member with them.<br>• Problem-solve with them to help find a solution to help with responsibilities while they have the appointment
	Recent bereavement / other emotional life event	• Suggest re-arranging Introduction call to an easier time<br>• Remind them of the number they can call if want to speak to the team at a time that would suit them [give number: 0113 392 6688]<br>• Emphasise flexibility of scheduling LHC appointment – we can make appointment at a time that suits them
**Communication difficulties – understanding/health literacy**	Can't read or understand the invitation letter clearly	• Offer to read the information in the letter to them over the phone/explain the LHC offer

		• Ask if they have a family member or friend who can help them read future letters received/if not explain they can always phone the number as happy to read to them<br><br>• **If appointment booked:** Inform them there will be a reminder call one week and one working day before the appointment.<br><br>• Explain that if they receive a letter and do not understand, they can call the number [give number: 0113 392 6688]
**Communication difficulties – non-Native English speaker**	Can't read or understand the invitation letter clearly	Offer to arrange call to be conducted through Language Line who can read information in the letter/leaflet to them<br>**If appointment booked:** Inform them there will be a reminder call one week and one working day before the appointment that can be conducted through LanguageLine.<br>Explain that if they receive a letter and do not understand, they can call the number [give number: 0113 392 6688]– can arrange call through LanguageLine
	During call	Offer to arrange call to be conducted through Language Line
	Worried about appointment	• Talk through step by step what would happen at appointment<br>• Suggest they arrange the appointment for a time when someone can come with them for support<br>• Arrange an interpreter to be available on the day.
**Communication difficulties – deaf/blind disability**	Can't read or understand letter / Can't communicate during call	• Offer to book a BSL or DeafBlind communicator who can read information in the leaflet/letter to them
	Worried about appointment	• Talk through step by step what would happen at appointment<br>• Suggest they arrange the appointment for a time when someone can come with them for support<br>• Arrange a BSL or DeafBlind communicator to be available on the day.

		• Confirm that a guide dog would be able to come to the van
Practical difficulties		
**Travel to screening**	Takes too long to get to screening location	• Check distance and book appointment at a time when van is at a location that is easiest for them to get to<br>• Emphasise that we can arrange the time of the appointment to be flexible to suit them<br>• Explain there are refreshment/toilet facilities nearby
	No/limited public transport options	• Consider asking family or a friend to take them to the appointment<br>• Emphasise that we can arrange the time of the appointment to be flexible to suit them<br>• Remind them of the number they can call if they need to cancel/re-arrange
	No-one available to take them there and/or back	• Emphasise that we can arrange the time of the appointment to be flexible to suit them<br>• If all other options have been exhausted, and there is no other way for them to attend the appointment, offer a taxi and help book this for them
	Potential cost / can't afford travel	• Consider asking family or a friend to take them to the appointment<br>• Explain there will be free parking available at the mobile van location<br>• If all other options have been exhausted, and there is no other way for them to attend the appointment, offer a taxi and help book this for them
	Worries about parking	• Explain there will be free parking available at the mobile van location<br>• Explore possible public transport routes and help plan their journey
	Mobility issues	• Explain the mobile vans are in easily accessible locations that the unit has a lift and has wheelchair access

		• Suggest they can bring a family member or friend with them for support<br>• If all other options have been exhausted, and there is no other way for them to attend the appointment, offer a taxi and help book this for them
**Comorbidities or related treatments**	Preventing undergoing screening (e.g., that being scanned could interfere with existing condition or treatment)	• Explain that the eligibility check is designed to ensure only people that can be safely scanned are invited to the LHC<br>• If the individual is still worried, suggest they can talk to a Senior Nurse or clinician or that they could contact their GP/treating doctor for peace of mind. Arrange the call with Senior Nurse/Clinician for them if this is their chosen option.<br>• Remind them it is entirely their decision.
	Variable health/clashing appointments or Comorbidities/related treatments take priority	• Emphasise flexibility of appointment<br>• Remind them of the number they can call if they need to cancel/re-arrange [give number: 0113 392 6688]<br>• Explain the importance of the LHC alongside competing comorbidities
	Preventing travel to screening	*See solutions for 'travel to screening'*
Psychological and social barriers		
**Already undergone relevant scan so believes LHC not needed**	Already undergone medical imaging/scans (other than chest CT)<br>or<br>Already have regular check-ups for respiratory condition (e.g., COPD)	• Explain the types of tests scan at a LHC (chest CT Scan), how this is different from the other imaging/scans they have already had, and why it is important (and that GP will be informed so oversight of all ongoing tests/care)
**Taking part is too much effort**		• Explain purpose and potential benefits of screening<br>• Explore if willing to discuss specific challenges that make it seem like too much of an effort, and problem-solve to find solution
**Lack of social support / social network**	Family/friends not supportive<br>Anxiety of family/friends	• Remind them they can bring a family/friend member with them to LHC if they think they could benefit from hearing more and

		that it is a 'no obligation' appointment<br>• Offer a telephone call that includes the individual and one of their respective family/friend over loud speaker<br>• Explore how the lack of support from family/friends is impacting their decision<br>• Explain that it is entirely their decision<br>• Explain purpose and importance of screening<br>• Offer a follow-up telephone call if the individual wants to speak again with friends/family before talking more with YLST team
**Worried will be made to stop smoking/ unwilling to stop smoking**	Worried about negative views/perceived blame others hold	• Explain there will be no judgements on smoking and the idea is to support those who want support (e.g., acknowledgement of generation when harms less well known, and physical addiction)
	Self-stigma and self-blame associated with smoking and lung cancer risk	• Explain there will be no judgements on smoking<br>• Discuss benefits of early diagnosis (that exist regardless of smoking status, age, current health status)
	Worried they will be made to stop smoking	• Explain that attending LHC does not mean they will be made to stop smoking
	Feels screening is pointless because cannot/does not want to stop smoking	• Discuss benefits of early diagnosis (that exist regardless of smoking status, age, current health status)<br>• Explain that if they would like to, they can choose to receive support from study to help them stop smoking (focus on efficacy)
**Fear, anxiety or worry about scanning/screening procedure**	Undergoing screening procedure itself<br>Claustrophobia about scanner	• Explain step-by-step what will happen at LHC appointment/during the screening procedure, including in the scanner<br>• Suggest they can bring a family member or friend with them for support<br>• Explain the staff will be supportive and understand, and can book a longer appointment so less pressure and can still choose not to<br>• Explain the difference between a CT scan and an MRI scan

	About exposure to radiation	• Discuss modern type of CT scanner used (low radiation dose CT) and level of risk associated compared to potential benefit. Compare with equivalent sources (i.e., natural environment and x-ray).
**Fear, anxiety or worry about having lung cancer**	Finding out they have lung cancer<br>Finding out they have another lung disease<br>Going through lung cancer treatment in the event of a positive result	• Explain what the LHC looks for and possible outcomes (including their frequency)<br>• Explain the benefits of early detection and impact on outcomes<br>• Explain how treatment is simpler and more successful/ address any treatment misconceptions
**Fatalistic beliefs about lung cancer prognosis**	"If I have lung cancer, I will die"<br>Screening won't make any difference<br>Treatment won't make any difference	• Explain what a Lung Health Check is and that most people will find out their lungs are working ok, but some people might benefit from more tests or treatment.<br>• Discuss benefits and efficacy of early detection and treatment on outcomes
**Unwilling to have treatment for lung cancer**	Surgery or other non-surgical treatments	• Explore reasons for feeling unwilling or incapable of having treatment. This may link to another solution/action we can offer (e.g., misperceptions about treatment, fatalistic beliefs, mistrust of the healthcare system, competing priorities)<br>• Explain what a Lung Health Check is and that most people will find out their lungs are working ok, but some people might benefit from more tests or treatment.<br>• Explore whether they could feel differently once they have the result of the scan, and that the treatment options would depend on the outcome of the scan.
**Mistrust of healthcare system**	Personal previous bad experience<br>Friend/family/other previous bad experience	• Explore what aspect of previous bad experience is preventing them attending the LHC. This could link to another solution/action we can offer.<br>• Explain step-by-step what will happen at LHC appointment/during the screening procedure, including in the scanner

		• Suggest they can bring a family member or friend with them for support<br>• Explain what the LHC looks for and possible outcomes<br>• Explain the benefits of early detection and impact on outcomes
**Does not feel they are at risk of lung cancer**	Does not feel at risk of lung cancer	Explain risk factors for developing lung cancer
**Does not feel they could benefit from lung cancer screening**	Perceives they are too old to benefit<br>Perceives they have smoked too long to benefit<br>Perceives that their health is too poor to benefit	Discuss benefits of early diagnosis (that exist regardless of smoking status, age, current health status)<br>Explain there will be no judgements on smoking
**Other**		
**No barriers discussed**		

Key techniques to use during the telephone appointment

During the call, Navigators should use the following techniques to enable an individual to make an informed choice about taking part in the Lung Health Checks:

Motivational Interviewing

You should have completed a separate Motivational Interview (MI) training course, delivered by The Association for Psychological Therapies, in addition to this Pathway Navigation specific training. You should apply the principles of this training to your Introduction to Lung Health Check call.

What is Motivational Interviewing?

Motivational Interviewing is a communication strategy that "helps a person see where they want to go, and helps them get there, by identifying clear strategies." [4]

It can be defined as:

"A planned approach to motivating people to recognise and do something about their problems – particularly for those who have mixed feelings about changing. It is persuasive, not coercive – it allows a person to review in their situation without judgement, supporting them to make a change, but not arguing with them to do so."[5]

Core MI communication techniques[6]

---

[4] The Association for Psychological Therapies, "Motivational Interviewing, and how to use it effectively", course presented by Dr William Davies
[5] The Association for Psychological Therapies, "Motivational Interviewing, and how to use it effectively", course presented by Dr William Davies
[6] https://motivationalinterviewing.org/understanding-motivational-interviewing

O Open questions

A Affirmation (of strengths, efforts, past successes)

R Reflections (reflecting back to show listening + empathy)

S Summarising (ensures shared understanding, reinforcement)

Change talk: where appropriate, spend more time discussing change talk (talk in favour of change) than sustain talk (what is being said against change) as research has shown conversations with more change talk are more likely to result in successful behaviour change.

Knowledge exchange: Respect that both the Navigator and the participant have expertise in the situation, with information sharing acknowledged as a two-way street. The Navigator should listen and be responsive to understanding and learning from what the participant is saying.

Core MI processes:

The Navigator should engage the following processes to enable the individual to engage with the LHC process if they wish to do so:

Engaging	Focusing	Evoking	Planning
Establish productive working relationship through listening, empathy, reflection, affirmation, respect of autonomy	Agenda is negotiated to agree on shared purpose<br><br>Introduction to LHC	Navigator gently explores to understand 'why' for change – ambivalence is normalised.<br><br>Risks/benefits/ barriers discussion	Navigator supports person to develop a plan to perform change/action

Communication techniques

Simple language

The terminology used in healthcare can be confusing, especially during times that might be distressing (such as when somebody is worried about lung cancer). Try to use simple language as much as possible, explaining things as you would to a friend or family member. Remember for many people English is not their first language so try to speak clearly. If somebody does not understand something you say, instead of repeating it, ask yourself if there is a simpler way to explain.

The National Center for Health Marketing have produced a plain language thesaurus for health communications that you might find helpful to look up specific terms you find yourself using a lot: https://stacks.cdc.gov/view/cdc/11500/

Keep language simple
Avoid medical jargon
Avoid acronyms

Instead of…	Try…
Cessation	Stopping
Conduct	Do
Consult with	Ask
Cure	Make completely better
Decrease	Lower
Diagnose	Test
Disease	Illness
Effective	Works well
Pulmonary	Related to the lungs
Risk	Chance

Implementation intentions (action planning)

- Implementation intentions are planning strategies that can help people move from a plan to do something, to successfully doing it.
- A lot of the time people intend to be screened, but simply don't get round to it. In cervical screening, half of those who do not attend, say they want to.
- Setting implementation intentions involves identifying any barriers that might stop a person from completing an action they intend to take. Then, finding strategies to bridge that gap between the intention to act, and completing the action itself.
- As a Navigator, work with the individual to identify the barriers that have stopped them engaging in the past, and help them to think of ways to overcome those. Use the Barriers and Strategies list on Page 20-27 to guide you.
- Help them make a plan for how they will attend their appointment before you finish the call. Help them think through how they will remember to attend the appointment, how they will travel to the appointment, and what other arrangements they need to make to help them attend (e.g., time off work or other responsibilities).
- Using implementation intentions will help more inclined non-engagers to successfully have a Lung Health Check.

For example, people may say they had intended to book a Lung Health Check appointment, but that they forgot, or other things came up. Here the barrier may be forgetfulness. Encouraging that individual to add the Lung Health Check to their calendar during the phone call could make them less likely to forget to attend.

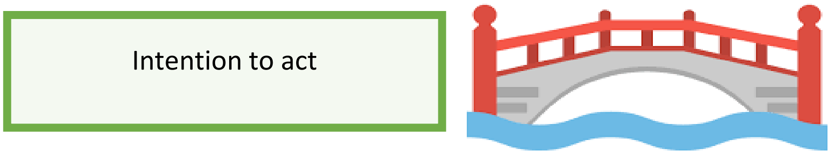

Teach back

- Teach back is a way of checking the information you are giving to an individual is being explained and understood clearly
- You cannot tell a person has understood simply by asking them 'Do you understand?'
- Instead, ask people to 'teach back' what you have just communicated to them.
- This is to check you have explained things clearly, not a test or assessment for the individual.
- If you notice the person is confused or explains incorrectly, you can try communicating the information again in a different way. For example, by using simpler language. Or, if English is not a person's first language, suggesting to re-arrange the call through a Language Line could help.
- For more information about Teach Back, visit the Health Literacy Place website https://www.healthliteracyplace.org.uk/toolkit/techniques/teach-back/

For example:

"So I can check I've explained things properly, can you tell me in your own words what we've discussed?"

"Just to check I have explained the process clearly, can you explain what you expect to happen at a Lung Health Check?"

"What are you going to do at the end of this call?" (put date of Lung Health Check appointment in diary / set up phone reminder)

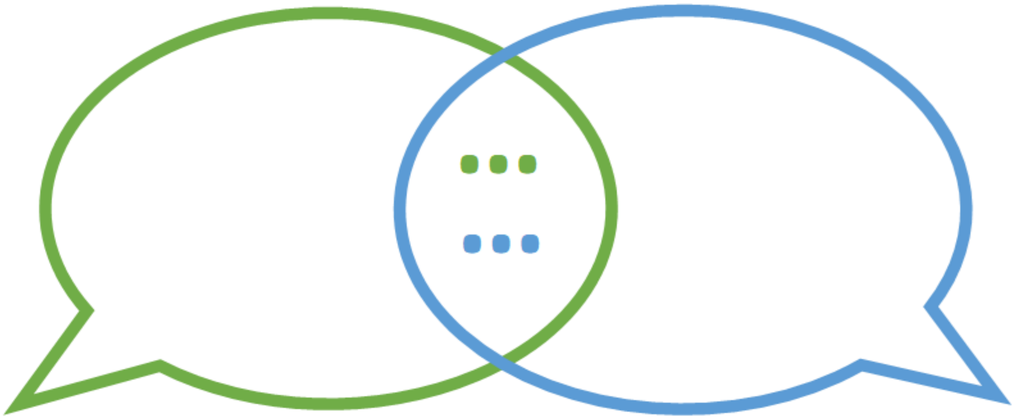

Receiving calls

When taking a call, it is important to understand why the individual is calling and what part of the Yorkshire Lung Screening Trial they are calling in relation to (i.e. which group of the study are they in):

- Are they calling in response to a letter they have received? The type of letter will help you understand how best to respond to the caller (see Table below). Although do bear in mind that individuals may not be able to accurately describe which letter they received.
- Are they calling following a previous call? Try to understand from the caller what previous contact they have had with the team.

Always request the individual's NHS number (preferred) or name/date of birth to search for their existing record, to see which group they have been assigned to.

Ensure each contact is recorded on PPM+. We need this information to work out the resource needed to deliver the pathway navigation intervention to evaluate its effectiveness.

Type of letter	Study group	Handling of the call
Individual calling about a letter with a **scheduled Introduction to Lung Health Checks telephone appointment**?	Pathway Navigation intervention group **or** Disengaged Responders	• Find out the reason for their call<br>• Handle with the principles of Pathway Navigation in mind.<br>• Be ready to work through the four stages of the Pathway Navigation call if the participant would like to conduct the Introduction to Lung Health Checks call at that time (see Page 38 for tip sheet)<br>• Record the contact made on PPM+ including completion of the Strategies and Barriers form
Individual calling about a **letter inviting them to book a Lung Health Check appointment**	Pathway Navigation control group **or**<br>T4 scan group	• Find out the reason for their call<br>• Handle as you would a standard query or appointment booking call for individuals not within the Pathway Navigation study
Individual calling about a letter with their **scheduled Lung Health Check appointment time**?	Pathway Navigation intervention group **or** Disengaged Responders **or** Pathway Navigation control group **or**<br>T4 scan group	• Find out the reason for their call<br>• Handle as you would a standard query call<br>• If within Pathway Navigation group, remember to complete the Strategies and Barriers form
Not calling in response to letter?		• Find out the reason for their call<br>• Determine which group they are in – if they are in a Pathway Navigation intervention group or Disengaged Responder group, handle with these principles in mind

Handling queries about the national lung cancer screening programme

You should not proactively raise the following information about the national lung screening programme during Pathway Navigation calls as it may confuse participants. However, if you are asked a question about how the Leeds Lung Health Checks fit with the national lung cancer screening programme, you can use this information to help answer any questions:

- There isn't a national lung cancer screening programme in the UK at the moment.
- However, over the past few years, there have been a number of studies and initiatives set up to test whether targeted lung screening could help reduce lung cancer deaths in the UK.
- The Lung Health Check programme being offered in Leeds through the Yorkshire Lung Screening Trial is one of these initiatives.
- Other initiatives, such as the NHS England Targeted Lung Health Check projects, are being offered in other areas of the country. All of these initiatives are offering the same lung cancer screening service to patients: an eligibility check, and a low dose CT scan if eligible.

The UK National Screening Committee (UK NSC) are currently reviewing data from lung screening trials across Europe and the UK. Over the course of the Leeds Lung Health Check Pathway Navigation study it is possible they will make a recommendation that national lung cancer screening should be implemented in the UK. If this happens, and a participant asks about it, you can reassure them that the Lung Health Check they receive through the Leeds programme is NHS-approved and will be the same as the NHS service being offered nationally.

SECTION 3: Activities to consolidate learning

TASK A: Using simple language

Use the spaces below to answer the following questions in simple language, like you would use during a Pathway Navigation call:

**What happens at a Lung Health Check?**

**Why would an individual have a lung health check?**<br>**What are the benefits? What are the risks?**

**What is the difference between a CT and MRI scan?**

TASK B: Case study discussions

Read through the case studies and discuss what you would say to each person during the Pathway Navigation phone call:

**Michael**
Michael is a Never Responder. You call him for the first time. He picks up the phone but is quiet and seems to be giving strange responses that don't quite make sense when you ask him if he understands why you have called. When you ask if he would be happy to have the eligibility check he says he does not know.
Notes:

**Chibunzo**
Chibunzo is a Never Responder. You see on her record there have been three attempts already at trying to call her. This is the fourth call. She picks up this time. She says she remembers getting the letters inviting her, but didn't have time to read the leaflet, and is sorry that she forgot the phone appointment. She is happy to go through the eligibility check but when you suggest booking an appointment she says she doesn't know her availability so says she will call back another time to make the appointment. She explains they only have one car between her and her partner, and he takes it to work, so she might not be able to get there at all.
Notes:

**Jay**
Jay is a Disengaged Responder. They apologise and explain that they had been planning on attending the Lung Health Check appointment the last time, but it all happened around the same time their partner had been diagnosed with cancer. There were lots of other appointments and they didn't feel they could cope with another one at the time. Their partner had been diagnosed late, was experiencing terrible treatment side effects and did not have a good prognosis. Jay mentioned they did not think they could cope if they also had to go through cancer and all the treatments, and that there wouldn't be a lot of point anyway – they'd smoked for years so they felt it was "just a matter of time."
Notes:

**Jay**
Radhika is a Never Responder. She already completed her Introduction to Lung Health Checks appointment two weeks ago, when she also made an appointment for her Lung Health Check. She has just telephoned to say she is sorry but she needs to cancel her appointment. She said she had a busy time coming up with work, her grandchildren were coming to stay soon, and she was feeling fit and healthy, so thinking about it she really just doesn't have time to attend the mobile van.
Notes:

TASK C: Practice Session - Role play

You can use this space to take notes during the role play practice session:

Pathway Navigation Call Tip Sheet

Good morning/afternoon, can I speak to [participant name] please?

My name is [your name]. I am calling from Leeds Teaching Hospital for your Introduction to Lung Health Checks telephone appointment. You should have received a letter about this appointment around 3 weeks ago.

Introduce the Lung Health Check Offer

Did they receive the appointment notification letter and leaflet?
Is now a convenient time to speak?
What do they already know about Lung Health Checks?
Explain Lung Health Checks: what, who for, what they involve
Risks and benefits of Lung Health Checks
CHECK UNDERSTANDING (TEACH BACK) AND GIVE OPPORTUNITY TO ASK QUESTIONS THROUGHOUT

Check risk-based eligibility

Full eligibility check (jnc Exclusion Criteria) for Repeat Non-Responders

Exclusion criteria only for Disengaged Responders

If eligible – book Lung Health Check appointment

Emphasise flexibility, prompt to consider travel arrangements and adding appointment to diary

Discuss barriers and strategies

Ask participants if they would be willing to take part in an in-depth interview at a later date (to understand individuals' experiences of the Pathway Navigation intervention)

During the call

Throughout the call record responses as prompted on PPM+

Use key communication skills: Motivational interviewing, Simple Language, Implementation Intentions, Teach Back

Keep in mind principles of informed consent

After the call

- Open the separate Barriers and Strategies form to record barriers/strategies discussed
- Complete and record follow up actions related to strategies (e.g., book taxi, book translator, record time for follow-up call

References and resources

Pathway Navigation

Harold P Freeman institute website: https://hpfreemanpni.org/

Motivational interviewing:
https://motivationalinterviewing.org/understanding-motivational-interviewing

Teach back:

Health Literacy Place: https://www.healthliteracyplace.org.uk/toolkit/techniques/teach-back/

Simple language thesaurus:

National Center for Health Marketing: **https://stacks.cdc.gov/view/cdc/11500/**

We would like to acknowledge the work of Dr Christian Van Wagner, Dr Lesley McGregor and their team, who developed a Patient Navigation manual as part of the Bowel Scope Screening Programme in Hull. The manual "Patient Navigation: Bowel Scope Screening Training Manual" formed the basis of this YLST Pathway Navigation training manual, which we adapted to the YLST setting.

Appendix 1. Barriers and Strategies PPM+ form categories

What barriers to engaging in screening were discussed?	1. Lack of awareness/knowledge about lung cancer and/or lung cancer screening<br>2. Avoidance of information about lung cancer, screening or lung cancer risk<br>3. Forgetfulness<br>4. Procrastination /failure to plan to make appointment<br>5. Competing priorities<br>6. Communication difficulties - language/health literacy<br>7. Communication difficulties - non-Native English speaker<br>8. Communication difficulties - deaf/blind disability<br>9. Travel to screening<br>10. Comorbidities or related treatments<br>11. Already undergone relevant scan so believes LHC not needed<br>12. Taking part is too much effort<br>13. Lack of social support / social network<br>14. Worried will be made to stop smoking / unwilling to stop smoking<br>15. Fear/anxiety/worry about scanning/screening procedure<br>16. Fear/anxiety/worry about having lung cancer<br>17. Fatalistic beliefs about lung cancer prognosis<br>18. Unwilling to have treatment for lung cancer<br>19. Mistrust of healthcare system<br>20. Does not feel they are at risk of lung cancer<br>21. Does not feel they could benefit from lung cancer screening<br>22. Other (please describe)<br>23. No barriers discussed
If other - please specify	Free text

What strategies to overcome the barriers to engaging in screening were discussed?	1. Explain what lung cancer is, risk factors and potential consequences<br>2. Explain purpose and potential benefits of lung cancer screening<br>3. Explained screening process step-by-step (length of LHC appointment, location, what happens, type of scan) and possible outcomes<br>4. Address lung cancer treatment concerns / misconceptions – discuss benefits of early diagnosis and treatment on lung cancer outcomes (that exist regardless of smoking status, age, current health status)<br>5. Reassurance about safety of screening process<br>6. Checked and corrected contact details (postal address / preferred phone number)<br>7. Re-arranged Introduction call to an easier time<br>8. Offered a follow-up phone call<br>9. Informed them of reminder call one week and one working day before the appointment.<br>10. Encouraged contact / provided number to call if they receive a letter that they do not understand or need to re-arrange telephone or LHC appointment<br>11. Suggested adding appointment to diary/calendar<br>12. Supported in planning travel arrangements (e.g. planned route, explain free parking at van)<br>13. Booked taxi to/from LHC appointment<br>14. Emphasised Introduction and LHC appointment flexibility (e.g. time that suits them, able to re-arrange)<br>15. Explained they can bring a friend/family member with them to support (in a carer capacity)<br>16. Held Introduction phone call over speaker phone<br>17. Problem-solve with them to help find a solution to help with responsibilities while they have the appointment<br>18. Arrange to conduct call and/or LHC appointment using LanguageLine or BSL/Deafblind interpreter<br>19. Confirm that a guide dog would be able to come to the van for the LHC<br>20. Explained the mobile vans are easily accessible, have a lift and wheelchair access, with toilet facilities nearby<br>21. Explained position on smoking (no judgements, not made to stop, but support offered to stop if they would like)<br>22. Explain self-determination (taking part is entirely their decision, no obligation, can change their mind)<br>23. Other (please describe)<br>24. No strategies discussed
If other - please specify	Free text
Is the participant willing to undergo a telephone interview about possible barriers?	Yes/No
