# Supplementary material for: Yorkshire Lung Screening Trial (YLST) pathway navigation study: a protocol for a nested randomised controlled trial to evaluate the effect of a pathway navigation intervention on lung cancer screening uptake
Source: BMJ Open. 2024 Jul 9;14(7):e084577. doi: 10.1136/bmjopen-2024-084577 (PMC11243133; doi:10.1136/bmjopen-2024-084577)
Supplement: online supplemental file 6 [file bmjopen-14-7-s006.pdf]

Queen Mary
University of London

Interview Topic Guide

The following questions and topics are intended as a rough guide and are not definitive.

Introduction for participants

The purpose of this interview is to explore your experiences and views of the 'Introduction to NHS Lung Health Check' telephone appointment you recently had, as well as barriers you might have experienced in accessing Lung Health Checks previously.

Interviewer will then talk through participant information sheet, answer any questions, remind the participant of their rights, and invite the participant to give verbal consent using the consent form and audio-recorder.

Note: Interviewer to clarify that while questions will include interactions with staff on the telephone, this will be completely anonymous and will not affect or be fed back to staff.

Overall acceptability/experience of scheduled telephone appointment approach

1. **Can you remember receiving the letter with your telephone appointment for the 'Introduction to NHS Lung Health Check'?**

- *Acceptability of concept - first thoughts/reactions/feelings*
- *Clarity/acceptability of appointment notification materials (appointment notification, leaflet, data statement)*
- *Concerns/feelings/expectations/thoughts about upcoming appointment*
- *Understanding/perception/perceived benefit of 'introduction' concept*
- *Suggestions for additional information or support at appointment notification stage*
- *Convenience/rescheduling of appointment*
- *Ease/difficulty of participating in telephone appointment as scheduled*

2. **Can you talk me through your experience of the telephone appointment?**

- *Overall perception of how the appointment/conversation went*
- *Openness/receptivity to telephone appointment*
- *Convenience/timing/privacy of appointment*
- *Range and acceptability of topics/discussion points covered*
- *Understanding of navigator's explanation of a Lung Health Check*
- *Perceptions of navigator's manner/approach/rapport/dynamic*
- *Experience of/reaction to lung cancer risk assessment/screening eligibility*
- *Ease of communicating with navigator/understanding*
- *Suggestions for additional informational/support to be provided during telephone appointment*
- *Discussions with friends/family/others afterwards*

Content and processes of change during the pathway navigation intervention

3. **Did the health professional you spoke to explore your views of Lung Health Checks and your thoughts about attending one?**

- *Acceptability of/receptivity to this topic*
- *Own perceived willingness/openness/receptivity to conversation about barriers to considering/attending a Lung Health Check*
- *Types of barriers/difficulties in attending shared/explored*
    - *Psychological capability: awareness/knowledge/planning/memory/language/literacy*
    - *Physical capability: health status/ability/transport*
    - *Opportunity: social support/location/norms/stigma/convenience/access/prompts*
    - *Motivation: concerns/fears/perceived lung cancer risk/screening risks and benefits/priority*
- *Strategies raised/used by navigator to address barriers/difficulties*
    - *Types of strategy <u>suggested</u>*
    - *Types of strategy <u>used</u>*
    - *Perceived usefulness/helpfulness of strategies*
    - *Receptivity to strategies*

4. **What, if anything, changed for you as a result of having the telephone introduction to a Lung Health Check?**

- *Thinking differently/motivation/planning - perceptions of lung cancer/lung cancer risk/screening/benefits/risks/relevance/understanding*
- *Previous problems or concerns lessened/worsened*
- *Improved/reduced opportunity – work or personal responsibilities/priorities/social support*
- *Physical capability: problem-solving/adjustments supporting access/language*
- *Implementation of specific strategies*
- *Effectiveness of specific strategies*
- *Hypothetical response if received usual care invitation/no telephone appointment*

5. **Did you have any further contact with the Lung Health Check team after the telephone appointment?**

- *One week and one day pre-appointment calls*
- *Attended Lung Health Check on mobile van?*
- *Other contact relating to arranging/facilitating specific strategies*
- *Views of further contact – helpful/burdensome/acceptable/necessary/receptivity*

History of previous engagement and reasons for non-response/disengagement

6. **Can you remember ever receiving an invitation to a Lung Health Check before?**

- *Recall of previous invitations (if none, skip)*
- *Thoughts/reactions/concerns following previous invitations*
- *Perceptions of risk/lung health/lung health checks*
- *Reasons for phoning/not phoning*

7. **(Disengaged group only) Can you talk me through your experience of talking to the Lung Health Check team on the telephone previously/attending the van?**

- *Thoughts/reactions/concerns during/following telephone eligibility assessment*
- *Experience/ease/receptivity related to communication during eligibility assessment*
- *Reasons for attending/not attending/not booking an in-person appointment at the van*
  - *Issues encountered during the telephone eligibility assessment*
  - *Psychological capability: awareness/knowledge/planning/memory/language/literacy*
  - *Physical capability: health status/ability/transport*
  - *Opportunity: social support/location/norms/stigma/convenience/access/prompts*
  - *Motivation: concerns/fears/perceived lung cancer risk/screening risks and benefits/priority*
- *Thoughts/reactions/concerns during/following in-person Lung Health Check appointment and/or CT scan at the van*
  - *Issues encountered during/following the appointment*
  - *Motivation to re-attend following results*

Interview close

8. **Finally, is there anything else you would like to share relating to your experience of the telephone appointment or Lung Health Checks?**

Interviewer to thank participant, answer any questions and provide with researcher contact details. Offer summary of findings in future if interested.
