# Supplementary material for: Yorkshire Lung Screening Trial (YLST) pathway navigation study: a protocol for a nested randomised controlled trial to evaluate the effect of a pathway navigation intervention on lung cancer screening uptake
Source: BMJ Open. 2024 Jul 9;14(7):e084577. doi: 10.1136/bmjopen-2024-084577 (PMC11243133; doi:10.1136/bmjopen-2024-084577)
Supplement: Supplementary file 7 [file bmjopen-2024-084577.reviewer_comments.pdf]

PEER REVIEW HISTORY

BMJ Open publishes all reviews undertaken for accepted manuscripts. Reviewers are asked to complete a checklist review form (**http://bmjopen.bmj.com/site/about/resources/checklist.pdf**) and are provided with free text boxes to elaborate on their assessment. These free text comments are reproduced below.

ARTICLE DETAILS

**TITLE (PROVISIONAL)**	The Yorkshire Lung Screening Trial (YLST) Pathway Navigation study: a protocol for a nested randomised controlled trial to evaluate the effect of a pathway navigation intervention on lung cancer screening uptake
**AUTHORS**	McInnerney, Daisy; Simmonds, Irene; Hancock, Neil; Rogerson, Suzanne; Lindop, Jason; Gabe, Rhian; Vulkan, Daniel; Marshall, Catriona; Crosbie, Philip; CALLISTER, Matthew; Quaife, Samantha

This article was not externally reviewed at BMJ Open. Protocols that have been independently assessed prior to submission to BMJ Open are usually fast-tracked to publication on the grounds that further substantial changes will not be possible. This independent assessment will usually be external, independent review for both a substantial grant award from a non-commercial or government funder and ethics approval.
